# Unveiling the Corrosion Mechanisms of High‐Entropy RETaO_4_ Through In Situ Observation

**DOI:** 10.1002/advs.202509828

**Published:** 2025-07-21

**Authors:** Zeyu Chen, Yiling Huang, Fan Peng, Chucheng Lin, Wei Zheng, Xuemei Song, Yaran Niu, Yi Zeng

**Affiliations:** ^1^ The State Key Lab of High Performance Ceramics and Superfine Microstructure Shanghai Institute of Ceramics Chinese Academy of Sciences Shanghai 200050 China; ^2^ Key Laboratory of Inorganic Coating Materials CAS Shanghai Institute of Ceramics Chinese Academy of Sciences Shanghai 200050 China

**Keywords:** CMAS corrosion, high‐entropy RETaO4, in situ observation, thermal barrier coatings

## Abstract

The ambiguous understanding of calcium‐magnesium aluminosilicate (CMAS) corrosion mechanisms in RETaO_4_ has hindered performance optimization through rare‐earth compositional engineering. This study systematically investigates the corrosion behavior of 3–10 component RETaO_4_ systems. In situ X‐ray diffraction/Transmission electron microscope coupled with Electron backscatter diffraction analysis unveils dynamic reaction pathways during the pre‐corrosion heating stage, identifying the crystallization and growth patterns of dominant corrosion product pyrochlore‐structured (Ca_2‐a‐b_RE_a_Al_b_)(Ta_2‐c‐d_Mg_c_Si_d_)O_7_. A reaction‐diffusion mechanism of CMAS corrosion for RETaO_4_ is proposed, highlighting the different behaviors of various rare earth elements in the corrosion process. Among eight types of RETaO_4_, La_1/6_Nd_1/6_Sm_1/6_Eu_1/6_Gd_1/6_Dy_1/6_TaO_4_ exhibits the best corrosion resistance, with a relatively thin corrosion layer and the ability to avoid element segregation and localized infiltration. These findings establish composition‐property relationships for designing next‐generation corrosion‐resistant thermal barrier coatings.

## Graphical abstract

Composition‐property relationships in multi‐component RETaO_4_ are unveiled. In situ XRD/TEM and EBSD expose dynamic crystallization pathways of the dominant corrosion product (pyrochlore) and reaction‐diffusion corrosion mechanisms. La_1/6_Nd_1/6_Sm_1/6_Eu_1/6_Gd_1/6_Dy_1/6_TaO_4_ emerges as the optimal composition, combining minimal corrosion layer thickness with suppressed localized infiltration.

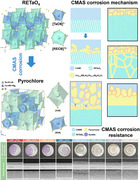

## 1 Introduction

Multifunctional thermal barrier coatings (TBCs) are gaining interest in gas turbine technology because they shield hot‐end components made of superalloys from thermal attack and environmental degradation in high‐temperature combustion environments.^[^ ^1^, ^2^ ^]^ The use of TBCs is essential for enhancing inlet gas temperature tolerance, engine power density, and fuel efficiency in advanced turbine systems.^[^ ^3^, ^4^, ^5^ ^]^ The conventional thermal barrier coating material, yttria‐stabilized zirconia (YSZ), suffers from phase transformation‐induced spallation at temperatures exceeding 1200 °C and exhibits relatively high thermal conductivity, which critically limits its applicability in next‐generation high thrust‐to‐weight ratio turbine engines requiring sustained operation above 1300 °C.^[^ ^6^, ^7^, ^8^ ^]^

Tantalate ceramics (RETaO_4_) have emerged as a leading candidate for next‐generation TBCs, owing to their exceptional combination of ultralow thermal conductivity (1.3 W m^−1^ K^−1^, 900 °C), tailored thermal expansion coefficients (9.06–10.01 × 10^−6^ K^−1^, 1200 °C), outstanding high‐temperature stability (>1400 °C), robust phase integrity under thermal cycling, and intrinsic ferroelastic toughening mechanisms that effectively suppress crack propagation.^[^ ^9^, ^10^, ^11^, ^12^, ^13^ ^]^ In addition to thermomechanical properties, corrosion resistance against calcium‐magnesium aluminosilicates (CMASs) is also a key criterion for evaluating TBC materials.^[^ ^14^, ^15^, ^16^ ^]^ Silicate environmental deposits formed from environmental dust, airborne sand, volcanic ash in aircraft engines, runway debris, and fly ash in power generation engines are commonly known as CMASs.^[^ ^17^, ^18^, ^19^ ^]^ CMAS corrosion is a major threat that contributes to TBCs failure.^[^ ^20^ ^]^ However, current research on CMAS corrosion of RETaO_4_ ceramics lacks consensus regarding both corrosion products and underlying mechanisms. Wu et al. identified M’‐YTaO_4_ and apatite Ca_0.135_Y_0.449_Ta_0.081_Si_0.336_O_1.683_ as primary CMAS corrosion products in YTaO_4_.^[^ ^21^ ^]^ In contrast, Ye et al. reported Ca_2_Ta_2_O_7_, Y_2_Si_2_O_7_, and M’‐YTaO_4_ as dominant products.^[^ ^22^, ^23^, ^24^ ^]^ Tian's work further revealed complex solid solutions (Ca_2‐x_RE_x_)(Ta_2‐y‐z_Mg_y_Al_z_)O_7_ as key corrosion products.^[^ ^25^ ^]^ In terms of corrosion mechanisms, the dissolution‐reprecipitation mechanism remains widely accepted for CMAS interactions.^[^ ^26^, ^27^ ^]^ However, our prior studies on monosilicates propose that the reaction‐diffusion mechanism offers greater interpretability for coating degradation processes, which likely extends to RETaO_4_ systems.^[^ ^28^ ^]^ These ambiguous understandings in corrosion mechanisms critically constrain the rational design of RETaO_4_ through compositional optimization strategies. Furthermore, conventional characterization techniques predominantly provide post‐corrosion static analyses, lacking dynamic in situ insights into real‐time CMAS corrosion reactions–essential data for mechanistic elucidation.

In this study, we synthesized multicomponent RETaO_4_ ceramics (nRE_1/n_)TaO_4_ (*n* = 3–10) through stepwise incorporation of rare‐earth elements (REEs) (Eu, Gd, Dy, Ho, Er, Tm, and Yb) into a La‐Nd‐Sm‐based system, followed by systematic investigation of their CMAS corrosion behavior. The dynamic corrosion reactions during the pre‐corrosion heating stage were characterized in real time using in situ XRD and in situ TEM. Post‐corrosion microstructural analysis via transmission electron microscope (TEM)‐electron backscatter diffraction (EBSD)‐energy‐dispersive X‐ray spectroscopy (EDS) confirmed the formation of ordered pyrochlore‐structured (Ca_2‐a‐b_RE_a_Al_b_)(Ta_2‐c‐d_Mg_c_Si_d_)O_7_ as the primary corrosion product, while also revealing its crystallization and growth patterns. These findings conclusively establish an elemental diffusion‐dominated mechanism governing CMAS corrosion in RETaO_4_ systems. This work not only lays a robust foundation for developing thermal barrier coatings for harsh‐service applications but also provides a generalizable strategy for designing corrosion‐resistant materials through controlled element composition.

## 2 Results

### 2.1 Microstructure of the Uncorroded RETaO_4_ Blocks

**Figure 1:**
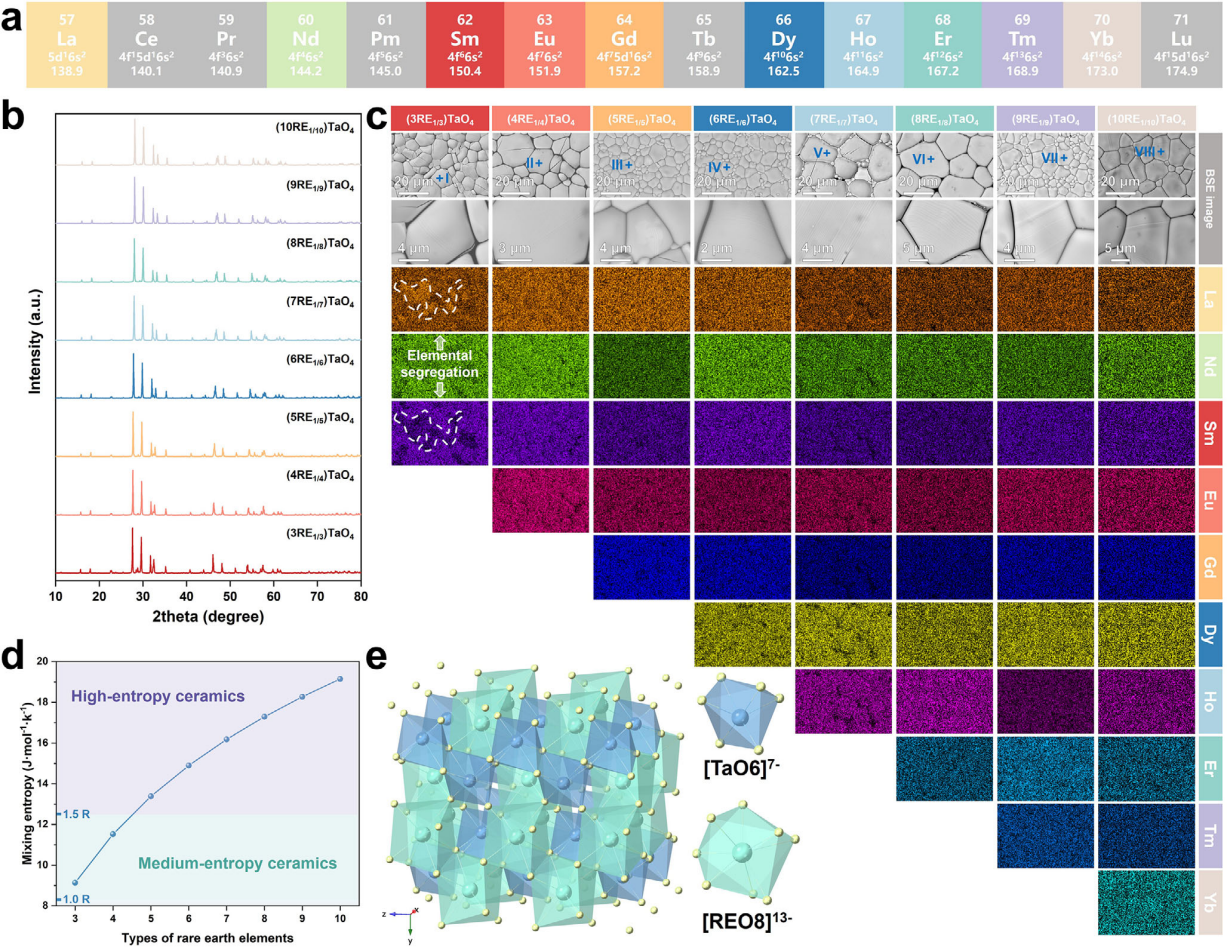
Phase structure and composition of eight types of RETaO_4_ blocks. a) The chosen lanthanide REEs, b) XRD patterns, c) SEM images and EDS elemental mappings, d) Relation between mixing entropy and numbers of elements, and e) Crystal structure of RETaO_4_.

**Figure** **1**a shows the 10 selected REEs from the lanthanide series used for the component design of RETaO_4_. We start with La, Nd, and Sm, which possesse the largest ionic radius among the ten REEs, and we progressively add elements with ionic radii close to Sm, which are supposed to gradually increase the mixing entropy. This approach led to the preparation of 3–10 component RETaO_4_ (La_1/3_Nd_1/3_Sm_1/3_TaO_4_, La_1/4_Nd_1/4_Sm_1/4_Eu_1/4_TaO_4_, La_1/5_Nd_1/5_Sm_1/5_Eu_1/5_Gd_1/5_TaO_4_, La_1/6_Nd_1/6_Sm_1/6_Eu_1/6_Gd_1/6_Dy_1/6_TaO_4_, La_1/7_Nd_1/7_Sm_1/7_Eu_1/7_Gd_1/7_Dy_1/7_Ho_1/7_TaO_4_, La_1/8_Nd_1/8_Sm_1/8_Eu_1/8_Gd_1/8_Dy_1/8_Ho_1/8_Er_1/8_TaO_4_, La_1/9_Nd_1/9_Sm_1/9_Eu_1/9_Gd_1/9_Dy_1/9_Ho_1/9_Er_1/9_Tm_1/9_TaO_4_, and La_1/10_Nd_1/10_Sm_1/10_Eu_1/10_Gd_1/10_Dy_1/10_Ho_1/10_Er_1/10_Tm_1/10_Yb_1/10_TaO_4_), named as (nRE_1/n_)TaO_4_ (where n represents the number of rare‐ earth (RE) components). The relationship between the mixing entropy and atomic fraction of elements is shown by the Equation (1).^[^ ^29^ ^]^

(1) $$\Delta S_{\mathrm{mix}}=k_{B}ln\Omega =-R\sum_{i=1}^{n}x_{i}lnx_{i}$$

Figure 1d demonstrates the relation between the mixing entropy and the number of REEs for an equiatomic mixture of elements calculated using Equation (1). When the number of REEs reaches 5 components or more, high‐entropy ceramics are formed.^[^ ^30^ ^]^ Figure 1b illustrates the XRD patterns of eight types of RETaO_4_, all of which exhibit the diffraction peaks of space group *I*2/*a*. The crystal structure of RETaO_4_ is shown in Figure 1e. The Ta atoms form [TaO_6_]^7‐^ polyhedron with six adjacent oxygen atoms, while the RE atoms form [REO_8_]^13−^ polyhedron with eight adjacent oxygen atoms. The surface morphology and EDS elemental mappings of eight RETaO_4_ blocks are presented in Figure 1c, where ferroelastic domains can be observed within the RETaO_4_ grains. Supplementary material (Table S1, Supporting Information) presents the RE element contents at various points in Figure 1c, which correspond to the theoretical values. Different REEs are evenly distributed, and there is no micron‐scale elemental segregation, except for (3RE_1/3_)TaO_4_. In (3RE_1/3_)TaO_4_, a significant elemental segregation phenomenon was observed. The mixing entropy obtained from the three REEs is low, which is insufficient to allow the elements to fully solidify into a single‐phase structure.^[^ ^31^ ^]^

### 2.2 In Situ Observation of Corrosion Reactions

**Figure 2:**
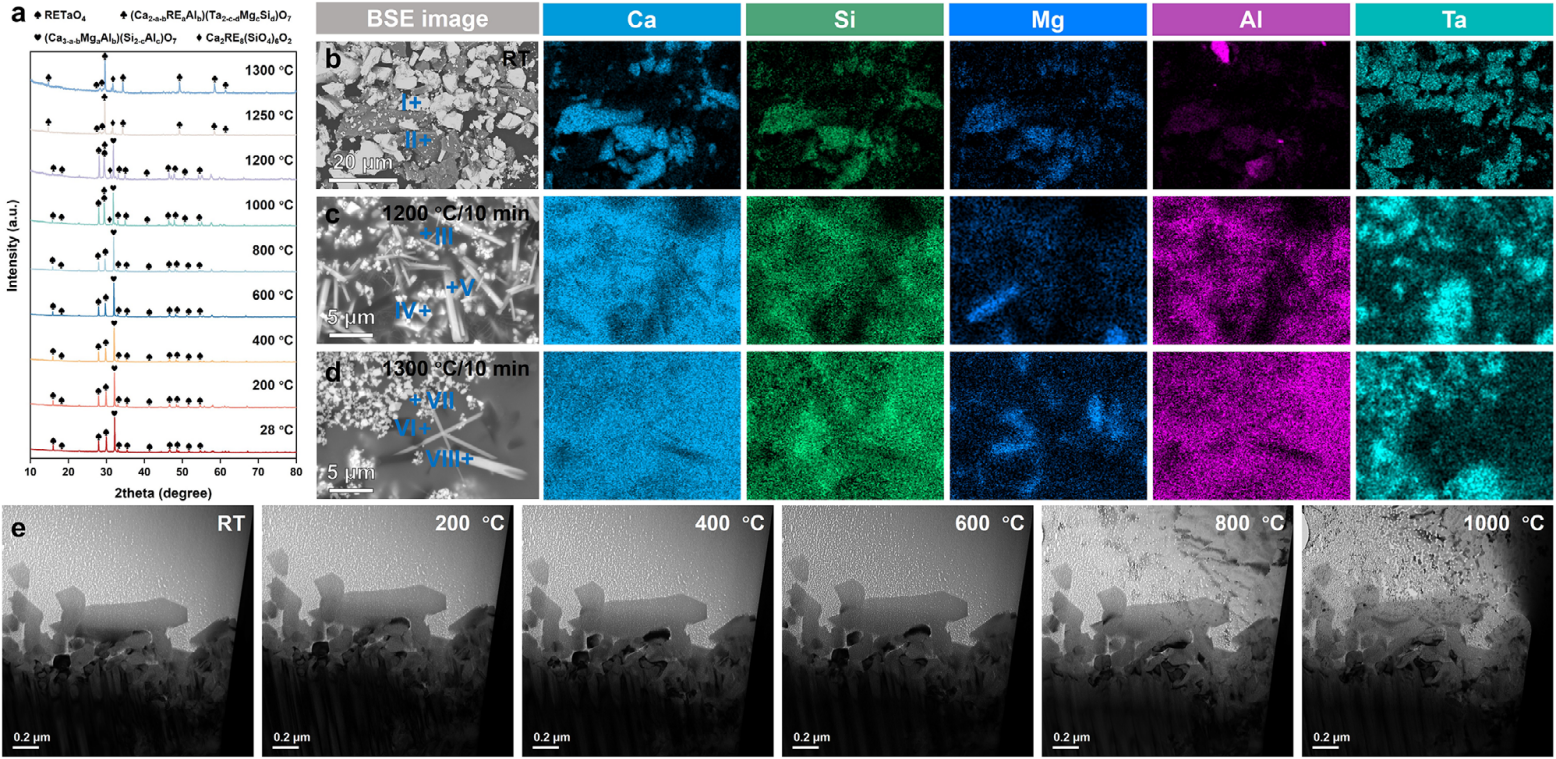
Structural and compositional characterization of (6RE_1/6_)TaO_4_ during in situ corrosion by CMAS. a) In situ XRD results of the mixed powders of CMAS and (6RE_1/6_)TaO_4_, b–d) The morphology and composition of the mixed powder at room temperature, after holding at 1200 °C for 10 min, and after holding at 1300 °C for 10 min, e) Overall morphological evolution of CMAS during in situ corrosion between RT and 1000 °C.

**Figure 3:**
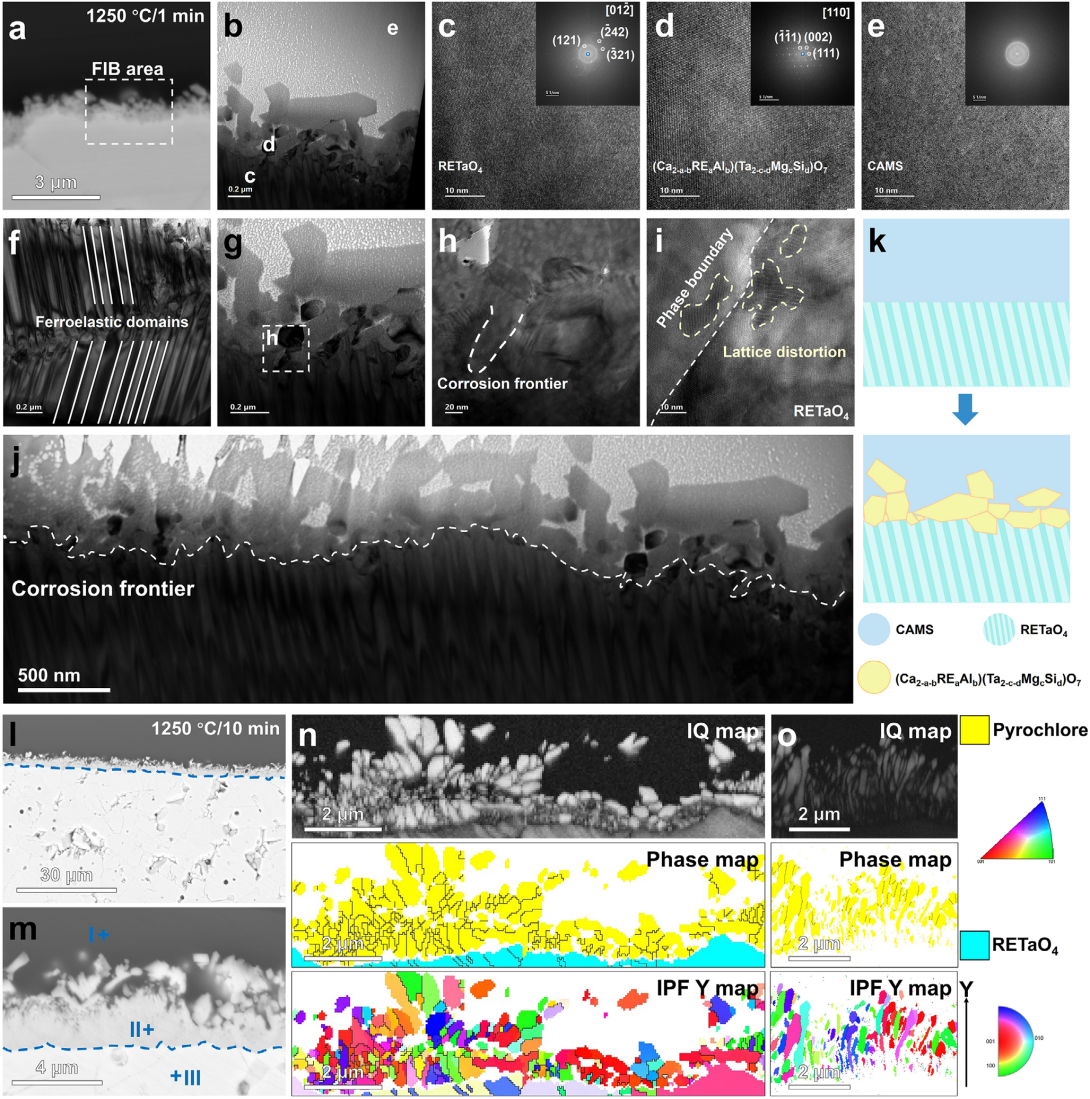
Nucleation mechanism of pyrochlore during CMAS corrosion initiation of RETaO_4_. a) Liftout area of TEM sample, b) Dark images, c–e) HRTEM images and SADE images of RETaO_4_, pyrochlore, and CMAS, f) Morphology of the ferroelastic domains of RETaO_4_, g–j) Corrosion frontier, k) Schematic illustration of pyrochlore nucleation, l,m) BSE images, n,o) EBSD results of newly nucleated pyrochlore grains.

Taking (6RE_1/6_)TaO_4_ as an example, the CMAS corrosion behavior of RETaO_4_ was investigated through in situ observations. Firstly, **Figure** **2**a shows the in situ XRD results of the mixed powders of CMAS and (6RE_1/6_)TaO_4_ within the temperature range from 28 to 1300 °C, simulating the reaction process of RETaO_4_ and CAMS during the heating. In addition to the diffraction peaks of RETaO_4_ shown in Figure 1, the mixed powder also exhibits a diffraction peak near 2θ ≈ 32°, which is generated by CMAS. In traditional understanding, CMAS is typically considered to have an amorphous structure, with its diffraction peak appearing as a broad peak, as shown in Figure S1 (Supporting Information). The diffraction peak near 2θ ≈ 32° may be generated by (Ca_3‐a‐b_Mg_a_Al_b_)(Si_2‐c_Al_c_)O_7_, formed through the self‐crystallization of elements in CMAS at low temperatures. Figure 2b–d shows the BSE images and EDS elemental mappings of the mixed powder at room temperature and after heat treatment at 1200 and 1300 °C. The compositions of the substance (I–VIII) with different contrasts are shown in Table S2 (Supporting Information). In Figure 2b, the substance I) with darker contrast represents the CMAS powder, while the substance II) with brighter contrast represents the (6RE_1/6_)TaO_4_ power, corresponding to the diffraction peaks in Figure 2a. Within the temperature range from room temperature to 800 °C, the diffraction peaks of mixed powers did not change. However, when the temperature increased to 1000 and 1200 °C, weak diffraction peaks from corrosion products apatite (Ca_2_RE_8_(SiO_4_)_6_O_2_) appeared. Additionally, the peak intensity in 2θ ≈ 30° significantly increased, which may be related to the formation of another corrosion product, pyrochlore ((Ca_2‐a‐b_RE_a_Al_b_)(Ta_2‐c‐d_Mg_c_Si_d_)O_7_). As shown in Figure 2c, after holding at 1200 °C, (6RE_1/6_)TaO_4_ reacted with CMAS III) to form corrosion products pyrochlore IV) and apatite V). However, at this temperature, CMAS has not completely melted and transformed into an amorphous phase. The characteristic peak of (Ca_3‐a‐b_Mg_a_Al_b_)(Si_2‐c_Al_c_)O_7_ is still clearly visible in Figure 2a. It was not until 1250 °C that the crystalline phase in CMAS completely disappeared. The diffraction peaks of the corrosion products gradually strengthened, with pyrochlore being the main corrosion product. As the temperature increased to 1300 °C, the diffraction peaks of the corrosion products continued to strengthen. The morphology of the molten CMAS VI) and the two corrosion products VII, VIII) after a 10 min reaction at 1300 °C is shown in Figure 2d. Figure 2e shows the in situ TEM results of the CMAS corrosion interface of (6RE_1/6_)TaO_4_, which was sampled from the area shown in Figure 3a using focused ion beam (FIB). CMAS, located at the top of the image, was identified as an amorphous phase by selected area electron diffraction (SADE) results (Figure 3e). In Figure 2e, from room temperature to 1000 °C (the temperature at which the corrosion reaction begins), CMAS shows some softening but has not yet transitioned into a liquid phase (see Video S1, Supporting Information). Therefore, before CMAS fully transitions into a liquid phase, the thermodynamic conditions for the corrosion reaction have already been met. This contradicts the traditional dissolution‐precipitation mechanism of CMAS corrosion on coating materials, where it is typically believed that CMAS corrodes coating materials by dissolving them and precipitating corrosion products.

### 2.3 Nucleation Mechanism of Pyrochlore

**Figure** **3** shows the nucleation process of the corrosion product pyrochlore during the initial CMAS corrosion stage of the (6RE_1/6_)TaO_4_ block at 1250 °C for 1 and 10 min. By combining the high‐resolution transmission electron microscopy (HRTEM) and SADE results shown in Figures 3c–e, it can be confirmed that, except for the CMAS with amorphous structure above in Figure 3b, the substance at the bottom of Figure 3b is (6RE_1/6_)TaO_4_, which exhibits the characteristic ferroelastic domains structure of the m‐type RETaO_4_ (Figure 3f). The nanoscale grains present between the CMAS and (6RE_1/6_)TaO_4_ are the corrosion products of pyrochlore. Figures 3g–i show the corrosion frontier at the interface between the corrosion products pyrochlore and (6RE_1/6_)TaO_4_, where lattice distortion regions are observed on both sides of the phase boundary. This may result from element diffusion and could indicate the onset of a phase transition of RETaO_4_ into pyrochlore.^[^ ^17^, ^25^ ^]^ As shown in Figure 3j, the corrosion frontier is formed by the interface between the continuous and uninterrupted pyrochlore isolation layer and (6RE_1/6_)TaO_4_, with no direct contact interface between CMAS and (6RE_1/6_)TaO_4_ was observed. Figure 3k illustrates the nucleation model of the pyrochlore grains during the early CMAS corrosion stage of RETaO_4_. After corrosion at 1250 °C for 10 min, the grain size of pyrochlore continues to increase, surpassing the resolution limit of the EBSD method. Therefore, EBSD was used to characterize the corrosion frontier. Figure 3l–o and Table S3 (Supporting Information) present the EBSD results along with the elemental composition of substances (I‐III) from Figure 3m. A continuous pyrochlore layer with a thickness of less than 5 µm forms, and the grains of pyrochlore become larger as the distance from the corrosion frontier increases. At the very front of the corrosion, the pyrochlore grains appear in elongated, tightly packed arrangements. Only at the early stages of corrosion, the direct contact between CMAS and RETaO_4_ is completely blocked, making it difficult for CMAS to dissolve RETaO_4_. Considering that the corrosion reaction begins before 1000 °C, the aforementioned phenomenon occurs before CMAS is fully melted. As shown in Figure S2 (Supporting Information), after corrosion at 1200 °C for 10 min, this dense barrier layer has already formed. Therefore, the corrosion of RETaO_4_ by CMAS occurs in the form of element diffusion, leading to the phase transition of RETaO_4_ to pyrochlore, rather than through dissolution‐reprecipitation.

### 2.4 Corrosion Mechanism of RETaO_4_ Over an Extended Period

**Figure 4:**
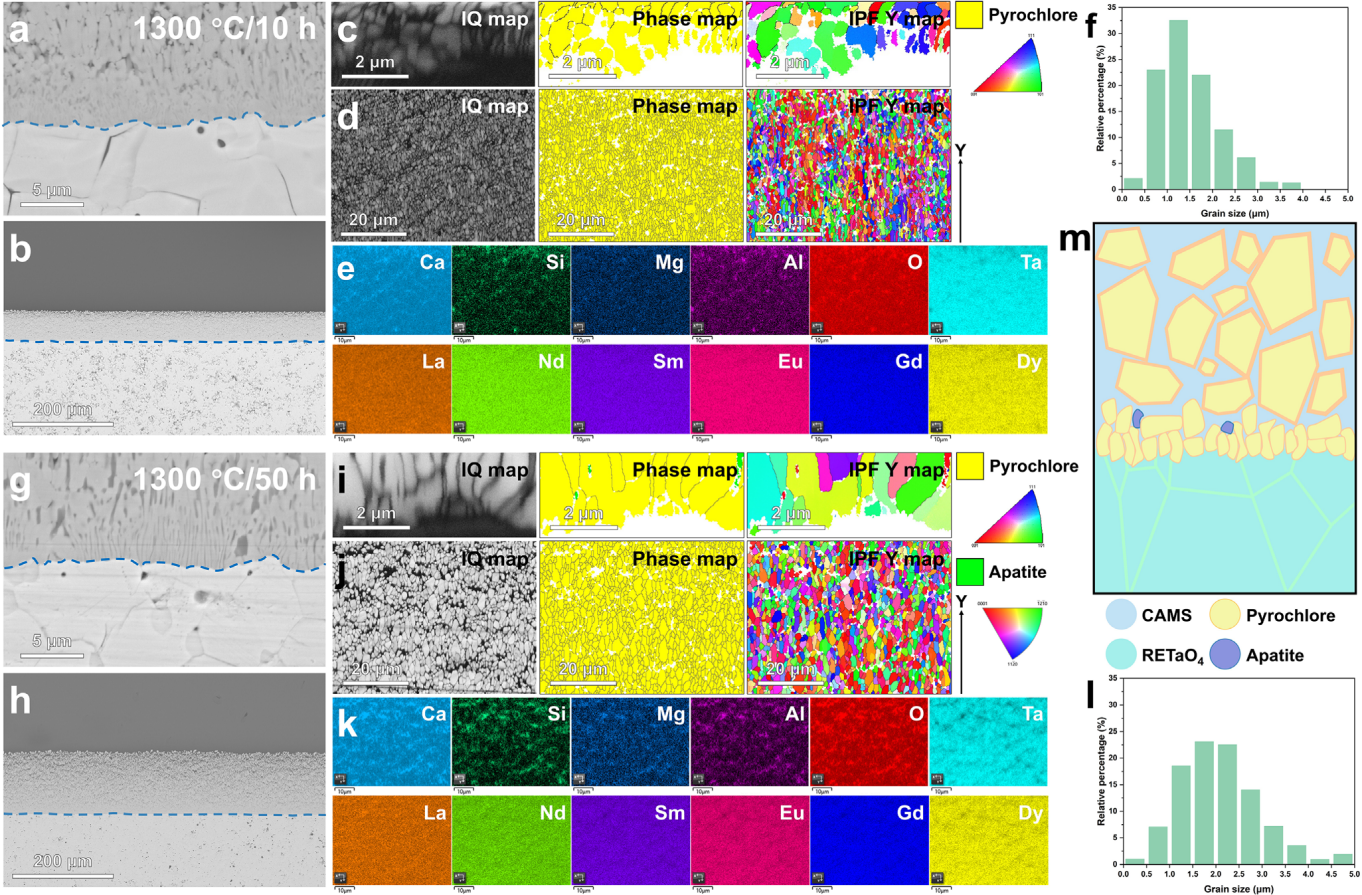
Corrosion results of RETaO_4_ at 1300 °C for 10 and 50 h. a,b) BES images of (6RE_1/6_)TaO_4_ corrode by CMAS for 10 h, c–e) EBSD and EDS results of corrosion result for 10 h, f) Grain size of pyrochlore formed after 10 h of corrosion, g,h) BES images of (6RE_1/6_)TaO_4_ corrode by CMAS for 50 h, i–k) EBSD and EDS results of corrosion result for 50 h, l) Grain size of pyrochlore formed after 50 h of corrosion, m) Schematic illustration of corrosion process.

With the increase in thrust‐to‐weight ratio, the intake temperature of next‐generation aircraft engines will gradually rise, and the service temperature of TBC will reach 1300 °C. Therefore, it is necessary to study the long‐term corrosion mechanism of RETaO_4_ at 1300 °C. **Figure** **4** shows the corrosion results of (6RE_1/6_)TaO_4_ at 1300 °C for 10 and 50 h. The corrosion layer at the CMAS and (6RE_1/6_)TaO_4_ interface is primarily composed of pyrochlore, with an average thickness of 58.5 µm (Figure 4b). The corrosion interface between pyrochlore and (6RE_1/6_)TaO_4_ is smooth and continuous (Figure 4a). Although CMAS (enriched regions of Ca and Si elements in Figure 4e) is present between the pyrochlore grains in the corrosion layer, no direct infiltration of CMAS along the RETaO_4_ block has been observed. The grain size of pyrochlore in the corrosion layer reaches the micron scale (Figure 4d,f), while at the corrosion frontier, the grain size of pyrochlore is smaller, mostly less than 1 µm (Figure 4c). As the corrosion time extends to 50 h, the average thickness of the corrosion layer increases to 115.1 µm, and the grain size of pyrochlore continues to grow (Figure 4j,l). However, the corrosion frontier remains a continuous interface between pyrochlore and RETaO_4_ (Figure 4h). The pyrochlore grains, with a size mostly smaller than 1 µm, form a continuous barrier layer that isolates CMAS from direct contact with RETaO_4_ (Figure 4g,i). Based on the corrosion results, combining the reaction‐diffusion theory, the corrosion mechanism of RETaO_4_ under prolonged corrosion can be inferred, as shown in Figure 4m: although the direct contact between RETaO_4_ and CMAS is blocked by pyrochlore, there still exists a chemical potential gradient across the CMAS→pyrochlore→RETaO_4_ direction. Elements in CMAS and RETaO_4_ will diffuse along this chemical potential gradient. When elements such as Ca and Si diffuse into the RETaO_4_ and exceed their solid solubility limits in RETaO_4_, a phase transformation from RETaO_4_ to pyrochlore will occur. Pyrochlore grains at the corrosion frontier are newly nucleated and have smaller grain sizes. As the corrosion frontier downward in the way of element diffusion and phase transformation, the grains at the old frontier gradually grow larger.

### 2.5 CMAS Corrosion Resistance and Corrosion Behavior of Different RETaO_4_

**Figure 5:**
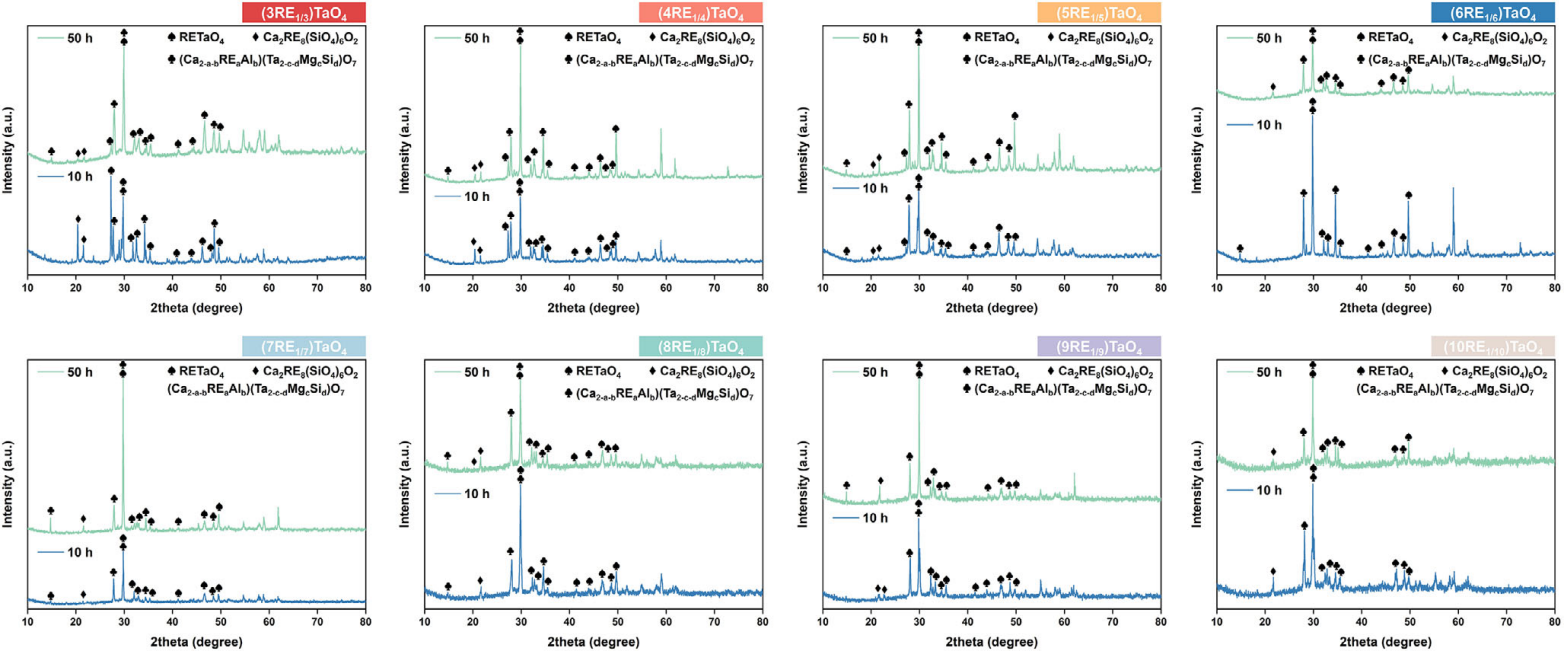
XRD patterns of CMAS post‐corroded surfaces of eight RETaO_4_.

The CMAS corrosion of eight types of 3–10 component RETaO_4_ was carried out at 1300 °C for 10 and 50 h. XRD patterns of the eight RETaO_4_ blocks corroded by CMAS are shown in **Figure** **5**. As shown, the diffraction peak on the block surface corresponded mainly to pyrochlore, with some apatite and RETaO_4_ peaks visible. The structure of pyrochlore is similar to Ca_2_Ta_2_O_7_, but unlike it, the Ca and Ta elements can be substituted by the REEs contained in the RETaO_4_ and the Mg, Al, and Si elements present in CMAS, making it a complex solid solution.

**Figure 6:**
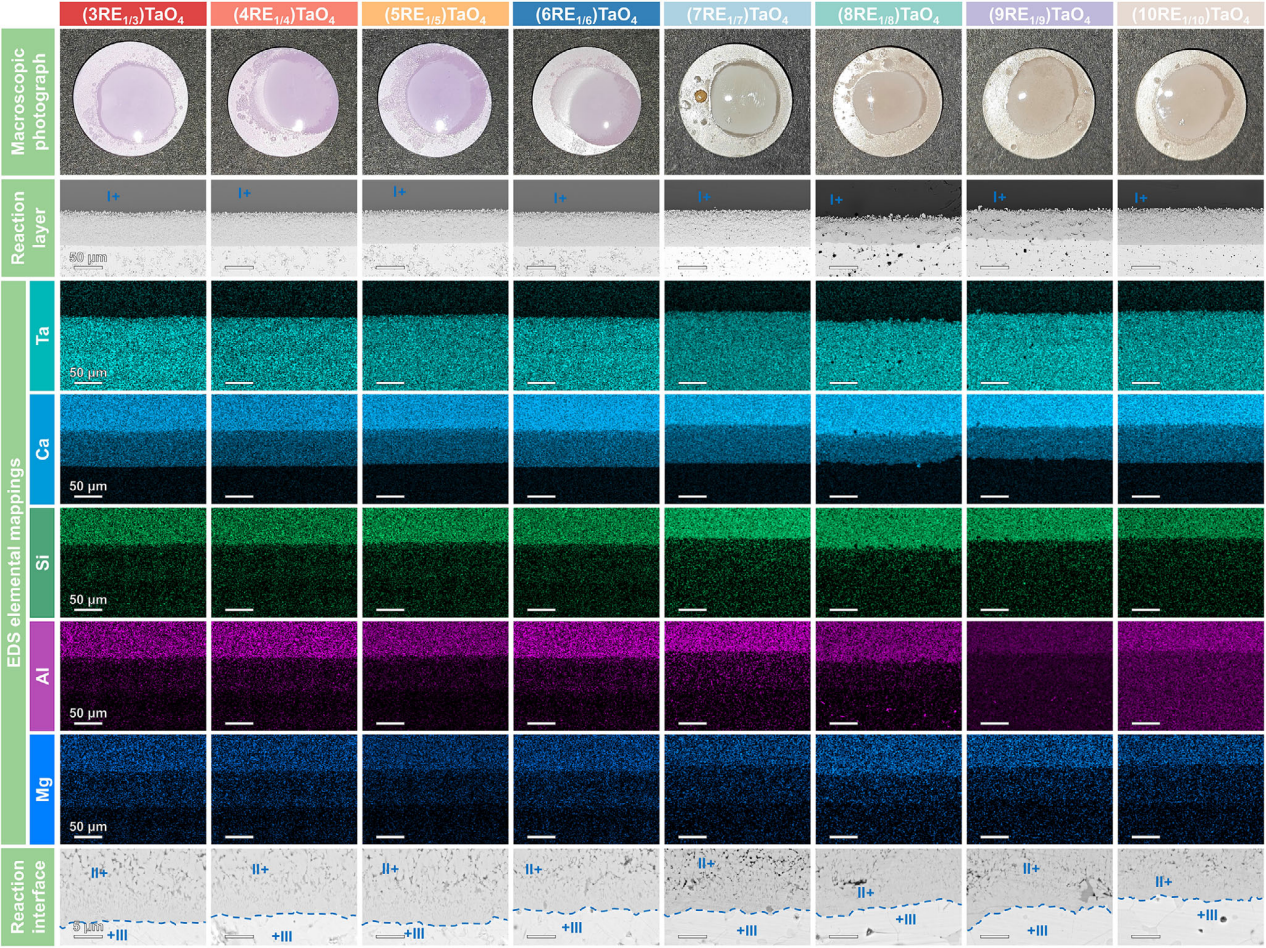
Macroscopic surface images, cross‐sectional morphology, and chemical element distribution of the eight RETaO_4_ blocks after corrosion at 1300 °C for 10 h.

**Figure** **6** shows the macroscopic surface images, cross‐sectional morphology, and EDS elemental mappings of the eight RETaO4 blocks after corrosion at 1300 °C for 10 h. According to the EDS semi‐quantitative analysis of points I‐III (at cross‐sectional morphology of different RETaO_4_) in the supplementary material (Table S4, Supporting Information), the substance (points I) covering the top of the blocks with the darkest contrast was the residual CMAS, corresponding to the brightest parts of the Si and Ca elemental mappings. The substance (points III) in the brightest contrast area at the bottom of the BSE images was uncorroded RETaO_4_. The grains (points II) accumulated at the interface between the CMAS and the RETaO_4_ blocks were the corrosion product pyrochlore, corresponding to the secondary brightest part of the Ca elemental mappings. Additionally, as shown in Figure S3 (Supporting Information), a small number of grains with a dark gray contrast corresponded to the apatite (corrosion product). Apatite formation is a common phenomenon during CMAS corrosion of RETaO_4_, however, the formation of apatite is very limited. A corrosion layer primarily composed of pyrochlore forms at the interface between CMAS and RETaO_4_. The average corrosion thickness of different samples is similar, ranging from 48.1 to 64.8 µm. The corrosion frontiers of eight samples were smooth and continuous, and no penetration of CMAS along the RETaO_4_ blocks was observed.

**Figure 7:**
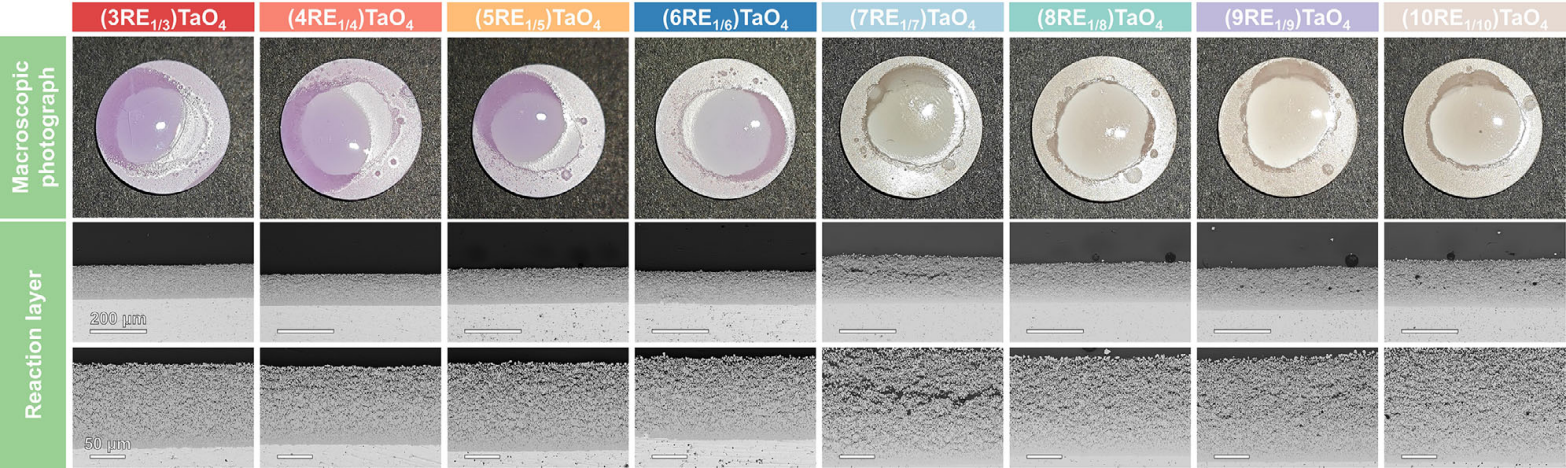
Macroscopic surface images and cross‐sectional morphology of the eight RETaO_4_ blocks after corrosion at 1300 °C for 50 h.

The corrosion results of the eight types of RETaO_4_ after 50 h of corrosion are shown in **Figure** **7**. The surface of the blocks still contains unreacted CMAS residues. However, with the increase in corrosion time, the average product layer thickness increases (ranging from 110.1 to 170.7 µm). In most of the samples, the corrosion frontier remains continuous and smooth.

**Figure 8:**
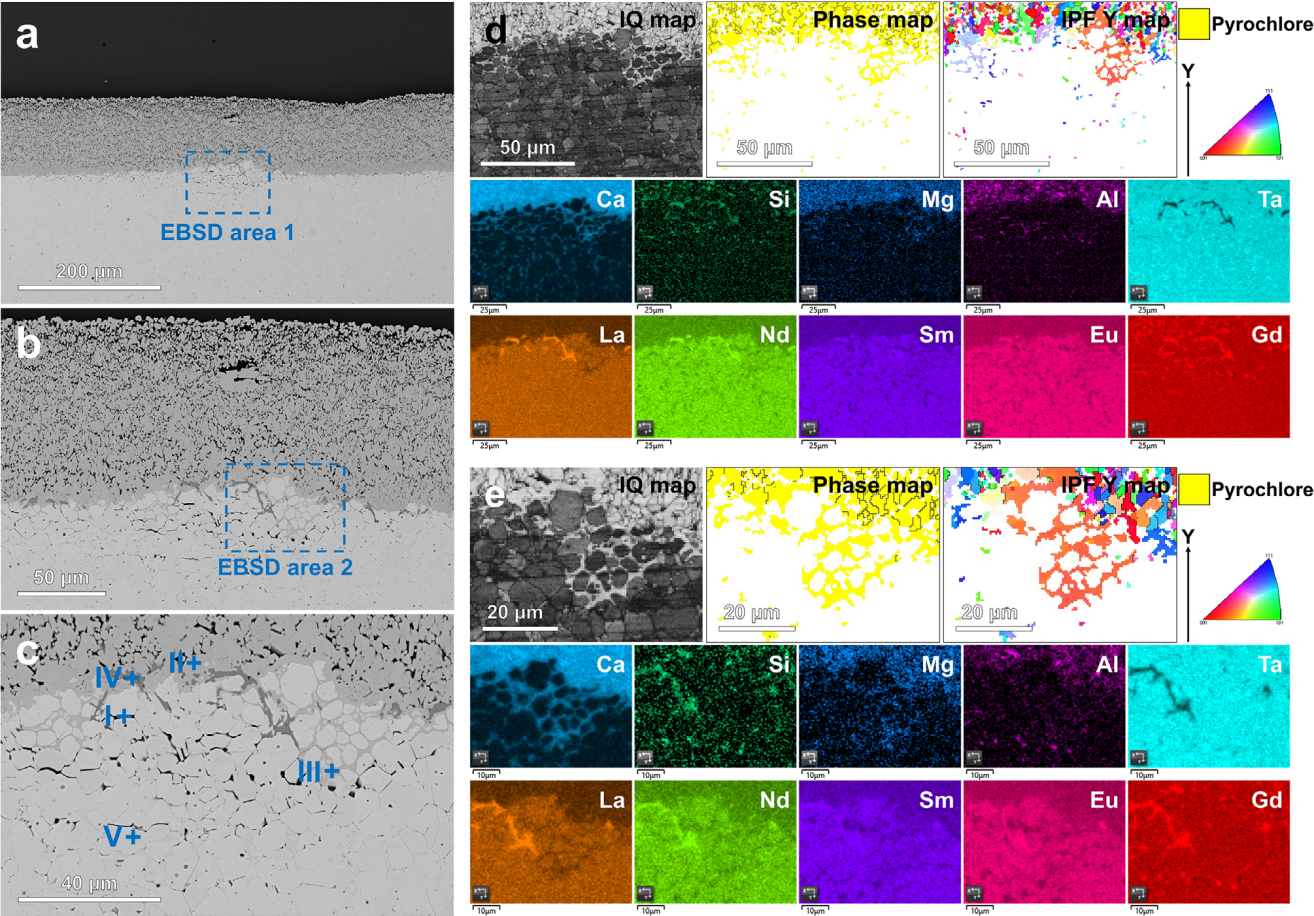
Corrosion results of (4RE_1/4_)TaO_4_ at 1300 °C for 50 h. a–c) BES images, d,e) EBSD and EDS results.

**Figure 9:**
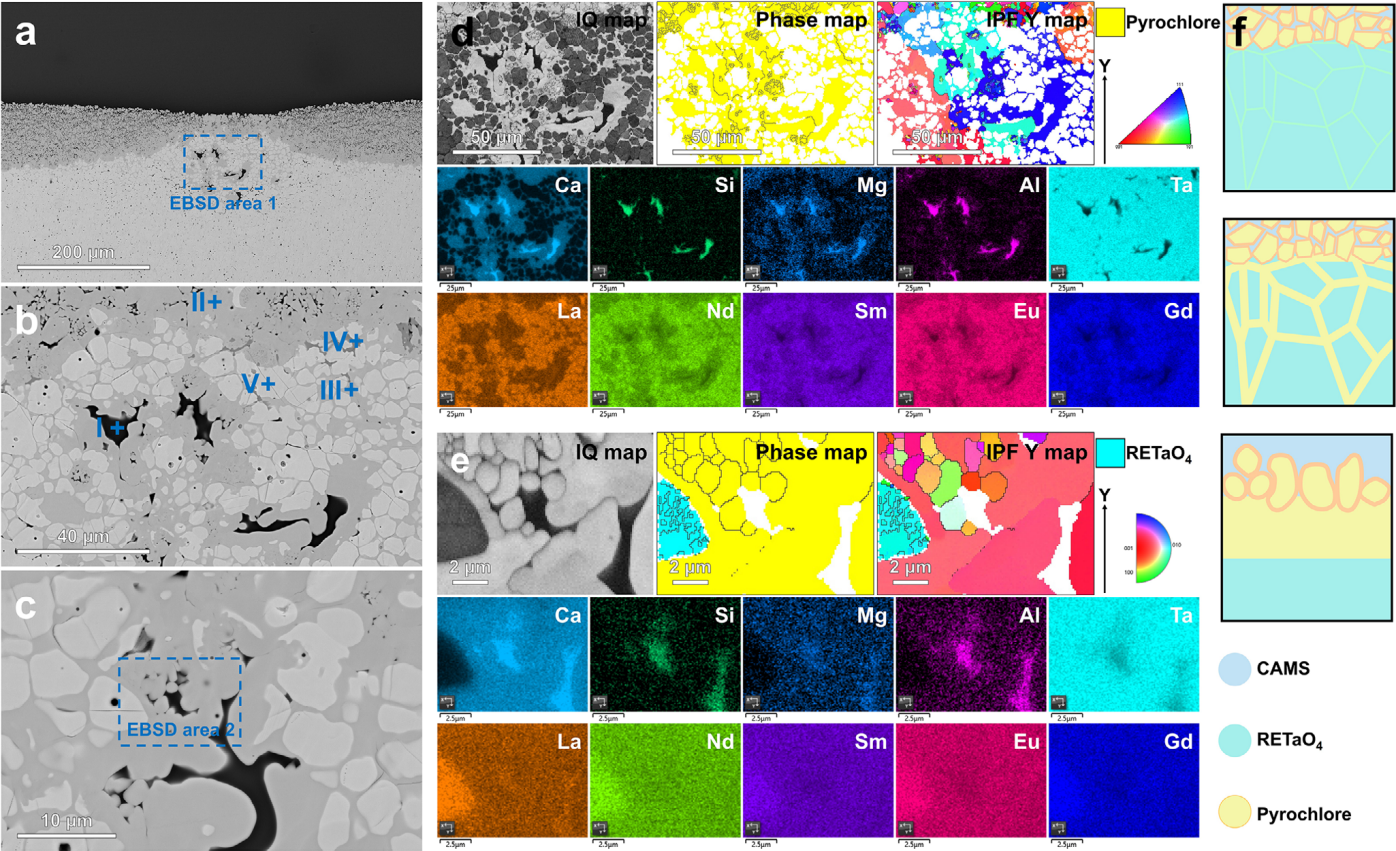
Corrosion results of (4RE_1/4_)TaO_4_ at 1300 °C for 50 h. a–c) BES images, d,e) EBSD and EDS results, f) Schematic diagram of the special growth and nucleation forms of pyrochlore.

However, in the corrosion results of (4RE_1/4_)TaO_4_ and (5RE_1/5_)TaO_4_, local penetration of CMAS and the presence of pyrochlore at the grain boundary of RETaO_4_ were observed. The local corrosion depths reached 153.1 and 170.4 µm, respectively, as shown in **Figures** **8**a–c and **9**a–c. The semi‐quantitative EDS analysis results for the substance (points in Figures 8c and 9b) are presented in Tables S5, S6 (Supporting Information). In the EBSD results shown in Figure 8d, an uneven corrosion frontier is observed. In addition to the presence of pyrochlore inside the RETaO_4_, a cobwebbed structure of pyrochlore extends from the corrosion layer into the RETaO_4_ direction at the corrosion frontier. The magnified view of this cobwebbed structure is shown in Figure 8e. In Figure 9d, the cobwebbed structure of pyrochlore is more prominent. Moreover, the adjacent regions of the cobwebbed pyrochlore show the same color in the IPF Y map, indicating that their grain orientations are similar.

Furthermore, the connection between the cobwebbed pyrochlore and the pyrochlore layer maintains the same orientation. This suggests that the pyrochlore did not precipitate as separate grains but rather grew along the grain boundaries of RETaO_4_ through elemental diffusion (Figure 9f). As shown in Figure 9e,f, in addition to this cobwebbed pyrochlore, pyrochlore at the interface between the cobwebbed pyrochlore and the penetrating CMAS exhibits another form with grain sizes mostly smaller than 2 µm and inconsistent grain orientations. The formation of these pyrochlore grains may also be related to elemental diffusion. On both sides of the cobwebbed pyrochlore, RETaO_4_ and CMAS exist, with various elements creating a chemical potential gradient. As elements like RE and Ta diffuse from the RETaO_4_ into the CMAS, pyrochlore precipitates when the elemental concentration in CMAS reaches nucleation conditions. Meanwhile, the elements from CMAS gradually diffuse toward the RETaO_4_ side, completing the phase transformation from RETaO_4_ to pyrochlore, i.e., the growth of pyrochlore.

## 3 Discussion

**Figure 10:**
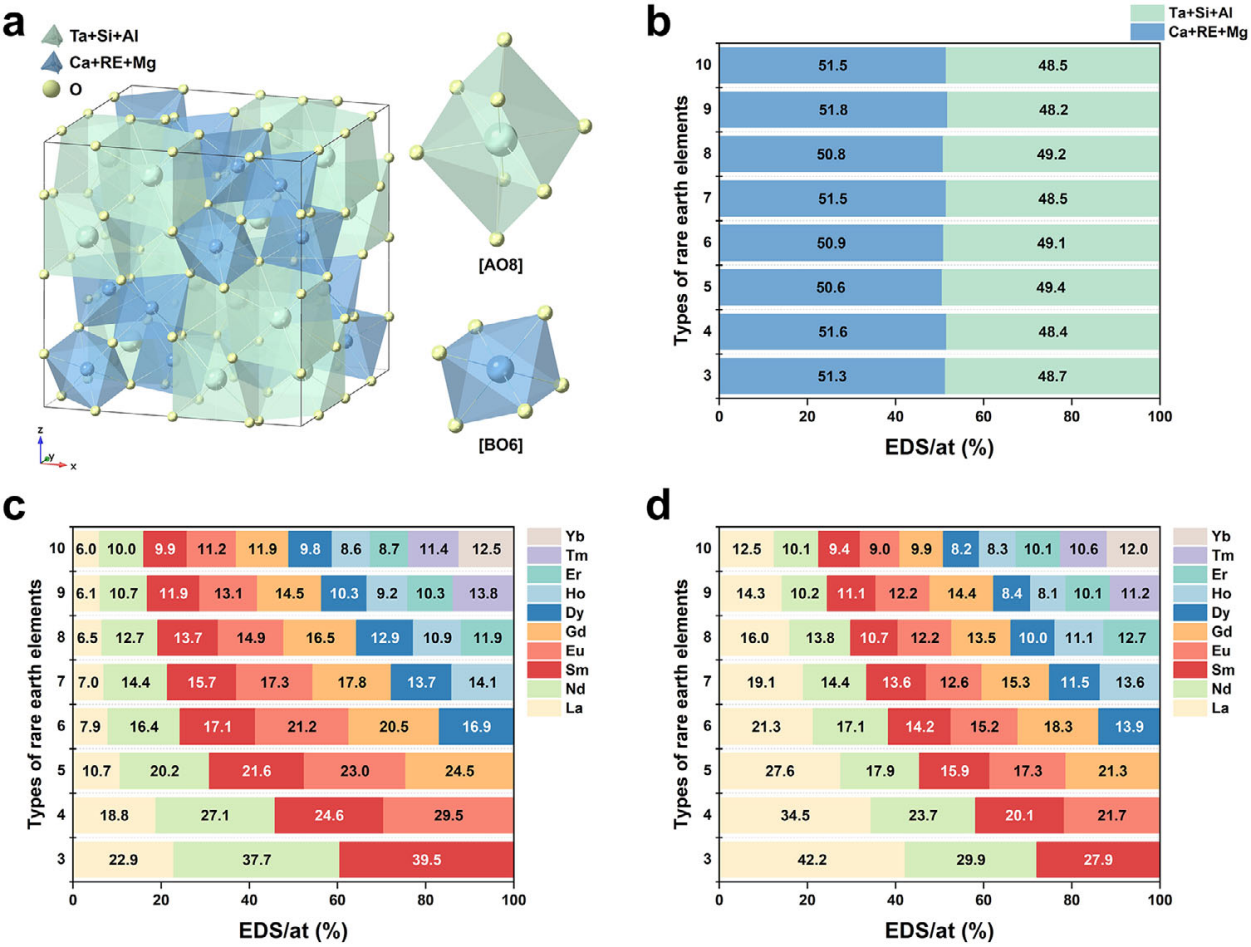
The corrosion behavior of different elements. a) The crystal structure of pyrochlore, b) the element content in pyrochlore, c) the RE elements content in pyrochlore, and d) the RE elements content in residual CMAS.

The crystal structure of the primary corrosion product (pyrochlore) formed in RETaO_4_ under CMAS corrosion conditions is shown in **Figure** **10**a. In this structure, cations occupy two distinct coordination environments: one coordinated with eight oxygen atoms forming [AO8] polyhedra, and the other coordinated with six oxygen atoms constituting [BO6] polyhedra.^[^ ^32^ ^]^ The EDS semi‐quantitative analysis results of pyrochlore formed from eight RETaO_4_ are presented in Table S7 (Supporting Information). The elemental composition reveals the presence of four elements (Ca, Mg, Al, and Si) from CMAS along with REEs and Ta elements originating from the RETaO_4_. The non‐random occupancy of these elements in the corrosion products is attributed to significant differences in their ionic radii, as summarized in Table S8 (Supporting Information).^[^ ^33^ ^]^ Elements with smaller ionic radii (e.g., Ta, Si, and Al) preferentially occupy the [BO6] sites, due to the lower coordination number and constrained chemical space at these positions. Conversely, elements with larger ionic radii (e.g., Ca, RE, and Mg) selectively occupy the [AO8] sites. Statistical analysis of the atomic percentages at A (Ca+RE+Mg) and B (Ca+RE+Mg) sites in corrosion products of eight RETaO_4_ is presented in Figure 10b. The combined atomic percentages of Ta, Si, and Al elements exhibit a near 1:1 ratio with the total content of Ca, RE, and Mg elements. This stoichiometric relationship validates the structural integrity and compositional accuracy of the corrosion product with an ordered pyrochlore structure in the cubic crystal system.

**Figure 11:**
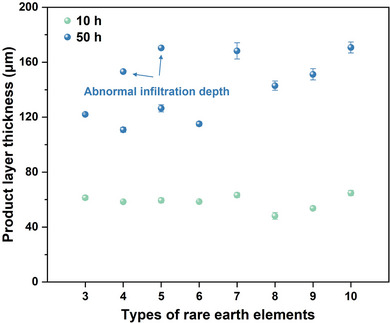
Average product layer thickness at different stages of corrosion.

Figure 10c,d illustrates the differential distribution of REEs between product pyrochlore and residual CMAS in eight RETaO_4_, with data calculated as the average of three spot measurements from Tables S7, S9 (Supporting Information). In apatite‐produced systems (e.g., equimolar multicomponent silicate, cerate, and so on), REE distribution follows an ionic radius‐dependent trend: larger REE^3^⁺ ions exhibit higher content in apatite, a phenomenon governed by the formation enthalpy of apatite.^[^ ^34^, ^35^, ^36^ ^]^ Contrastingly, pyrochlore demonstrates distinct REE partitioning behavior. The largest RE^3+^ ion (La^3+^, ≈1.16 Å) preferentially diffuses into CMAS melt rather than incorporating into the pyrochlore lattice, while medium‐sized ions (Sm^3^⁺ ≈1.08 Å, Eu^3^⁺ ≈1.07 Å, Gd^3^⁺ ≈1.05 Å) show significant enrichment in pyrochlore. This indicates that pyrochlore formation involves competing thermodynamic factors beyond simple ionic radius considerations. The reaction‐diffusion mechanism dominates the CMAS corrosion of RETaO_4_ at temperatures ≤1300 °C, making kinetic effects more critical than thermodynamic rules. Smaller REEs with faster diffusion rates lead to increased 50 h corrosion thickness as their component number rises, as shown in **Figure** **11**. The reason for the similar corrosion thickness during the initial 10 h of corrosion may be that the chemical element content difference between CMAS and RETaO_4_ is significant, creating a large chemical potential gradient, which allows for sufficient reaction between the two. Similar to the structure of pyrochlore, the structure of RETaO_4_ shown in Figure 1e is also composed of two types of polyhedra: [REO8]^13−^ and [TaO6]^7−^. Therefore, the phase transition of RETaO_4_ to pyrochlore can be understood as the diffusion of Ca and Mg from CMAS into the [REO8]^13−^ sites, while Al and Si from CMAS diffuse into the [TaO6]^7−^ sites of the RETaO_4_. Among these, the diffusion of Ca is the most critical. The average RE^3+^ radius in the four‐component (1.10 Å) and five‐component (1.09 Å) RETaO_4_ is quite close to that of Ca (1.12 Å), which facilitates the diffusion of Ca. This may explain the localized cobwebbed growth of pyrochlore observed in both RETaO_4_. Moreover, when the number of RE components (3) is relatively low, phenomena such as insufficient mixing entropy to form a single phase may occur. Therefore, the six‐component RETaO_4_ exhibits the optimal corrosion resistance performance.

## 4 Conclusion

In this study, we systematically investigated the CMAS corrosion behavior of multicomponent RETaO_4_. The primary corrosion product was identified as pyrochlore ((Ca_2‐a‐b_RE_a_Al_b_)(Ta_2‐c‐d_Mg_c_Si_d_)O_7_) accompanied by minor apatite (Ca_2_RE_8_(SiO_4_)_6_O_2_). Throughout the corrosion process, pyrochlore persistently served as an intermediate barrier, effectively preventing direct contact between CMAS and the RETaO_4_. Consequently, the CMAS corrosion of RETaO_4_ follows a reaction‐diffusion mechanism, where elemental diffusion governs both pyrochlore nucleation and the phase transformation from RETaO_4_ to pyrochlore. The six‐component La_1/6_Nd_1/6_Sm_1/6_Eu_1/6_Gd_1/6_Dy_1/6_TaO_4_ exhibits the most ideal corrosion resistance due to the following three reasons: First, it avoids the increased corrosion thickness caused by the small ionic radius of REEs. Second, it has sufficient mixing entropy to form a single phase. Third, it prevents the occurrence of local permeation phenomena.

## 5 Experimental Section

### Sample Preparation

Eight types of RETaO_4_ were prepared via a solid‐phase method. The starting materials consisted of RE_2_O_3_ (RE = La, Nd, Sm, Eu, Gd, Dy, Ho, Er, Tm, and Yb) and Ta_2_O_5_ powders. Each RE_2_O_3_ was mixed in equal molar ratios, with the total RE_2_O_3_ to Ta_2_O_5_ molar ratio fixed at 1:1. The powder mixture was homogenized in a vertical nylon tank using a ball mill with ethanol and zirconia balls as the dispersion medium. The resulting slurry was dried at 120 °C for 5 h and sieved. The sieved powders were cold‐pressed into circular molds (20 mm diameter) under 200 MPa pressure for 20 s. The green compacts were then sintered in a muffle furnace at 1500 °C for 5 h followed by 1600 °C for 10 h to obtain ceramic blocks.

CMAS powders were synthesized via the solid‐phase method with a Ca_33_Mg_10_Al_13_Si_44_ molar composition using CaO, MgO, Al_2_O_3_, and SiO_2_ as precursors. The stoichiometric mixture was ball‐milled in a vertical nylon tank for 4 h. The slurry was dried at 120 °C and sieved through a 20‐mesh sieve. The mixed powders were heated in a muffle furnace at 1400 °C for 4 h, cooled to 800 °C at 10 °C min^−1^, and then air‐cooled to room temperature to form a CMAS bulk, which was subsequently ground into powder.

### CMAS Corrosion Tests

The CMAS powder was blended with ethanol and uniformly applied onto the ceramic blocks. The CMAS concentration was calibrated to ≈30 mg cm^−2^ through iterative coating and drying cycles. The coated blocks were heated in a muffle furnace under an air atmosphere at 1200–1300 °C.

### Structure and Compositional Characterization

Corroded samples were cross‐sectioned, mechanically ground, and polished to a 2500‐grit finish. A scanning electron microscope (SEM, Verios G4, FEI, USA) equipped with an energy‐dispersive X‐ray spectrometer (EDS, Oxford, UK) and electron backscatter diffraction (EBSD, Oxford, UK) was employed to analyze the microstructural morphology, elemental distribution, and crystallographic information. Corrosion layer thickness was calculated as the average of five measurements, while elemental compositions in pyrochlore and CMAS were derived from three averaged data points.

For in situ heating TEM analysis, a TEM liftout specimen was extracted from the corroded cross‐section (Figure 3a) using an FEI focused‐ion beam (FIB) system. The sample was thinned to electron transparency via standard FIB protocols, including a final low‐energy cleaning step. The microstructure was characterized by transmission electron microscopy (TEM, Tecnai F20, FEI, USA) from room temperature to 1000 °C.

Phase compositions of all eight RETaO_4_ blocks before and after corrosion were analyzed by X‐ray diffraction (XRD, D8 ADVANCE, Bruker, Germany) with Cu‐K_α_ radiation (40 kV, 40 mA). Data were collected using a 0.6 mm divergence slit over two 2θ ranges: 10–110° (0.8° min^−1^ scan rate) and 10°–80° (10° min^−1^ scan rate). In situ high‐temperature XRD of mixed CMAS/(6RE_1/6_)TaO_4_ powders was performed with a heating stage, covering 10°–70° (5° min^−1^ scan rate) from room temperature to 1300 °C (10 °C min^−1^ heating rate, 15 min dwell time per temperature step).

## Conflict of Interest

The authors declare no conflict of interest.

## Supporting information



Supporting Information

Supplementary Video 1

## Data Availability

The data that support the findings of this study are available from the corresponding author upon reasonable request.
