# Supplementary material for: Unveiling the Corrosion Mechanisms of High‐Entropy RETaO4 Through In Situ Observation
Source: Adv Sci (Weinh). 2025 Jul 21;12(39):e09828. doi: 10.1002/advs.202509828 (PMC12533205; doi:10.1002/advs.202509828)
Supplement: Supplementary file 1 — Supporting Information [file ADVS-12-e09828-s002.docx]

Supporting Information

Title Unveiling the corrosion mechanisms of high-entropy RETaO_4_ through in-situ and ex-situ observation

Author(s), and Corresponding Author(s)*

Zeyu Chen, Yiling Huang*, Fan Peng, Chucheng Lin, Wei Zheng, Xuemei Song, Yaran Niu, Yi Zeng*

A Zeyu Chen, Yiling Huang, Fan Peng, Chucheng Lin, Wei Zheng, Xuemei Song, Yi Zeng*

B *Yaran Niu*

A The State Key Lab of High Performance Ceramics and Superfine Microstructure, Shanghai Institute of Ceramics, Chinese Academy of Sciences, Shanghai, 200050, China.

B Key Laboratory of Inorganic Coating Materials CAS, Shanghai Institute of Ceramics, Chinese Academy of Sciences, Shanghai, 200050, China.
E-mail: huangyiling@mail.sic.ac.cn (Yiling Huang), zengyi@mail.sic.ac.cn (Yi Zeng)

Figure S1. The XRD pattern of CMAS powder.


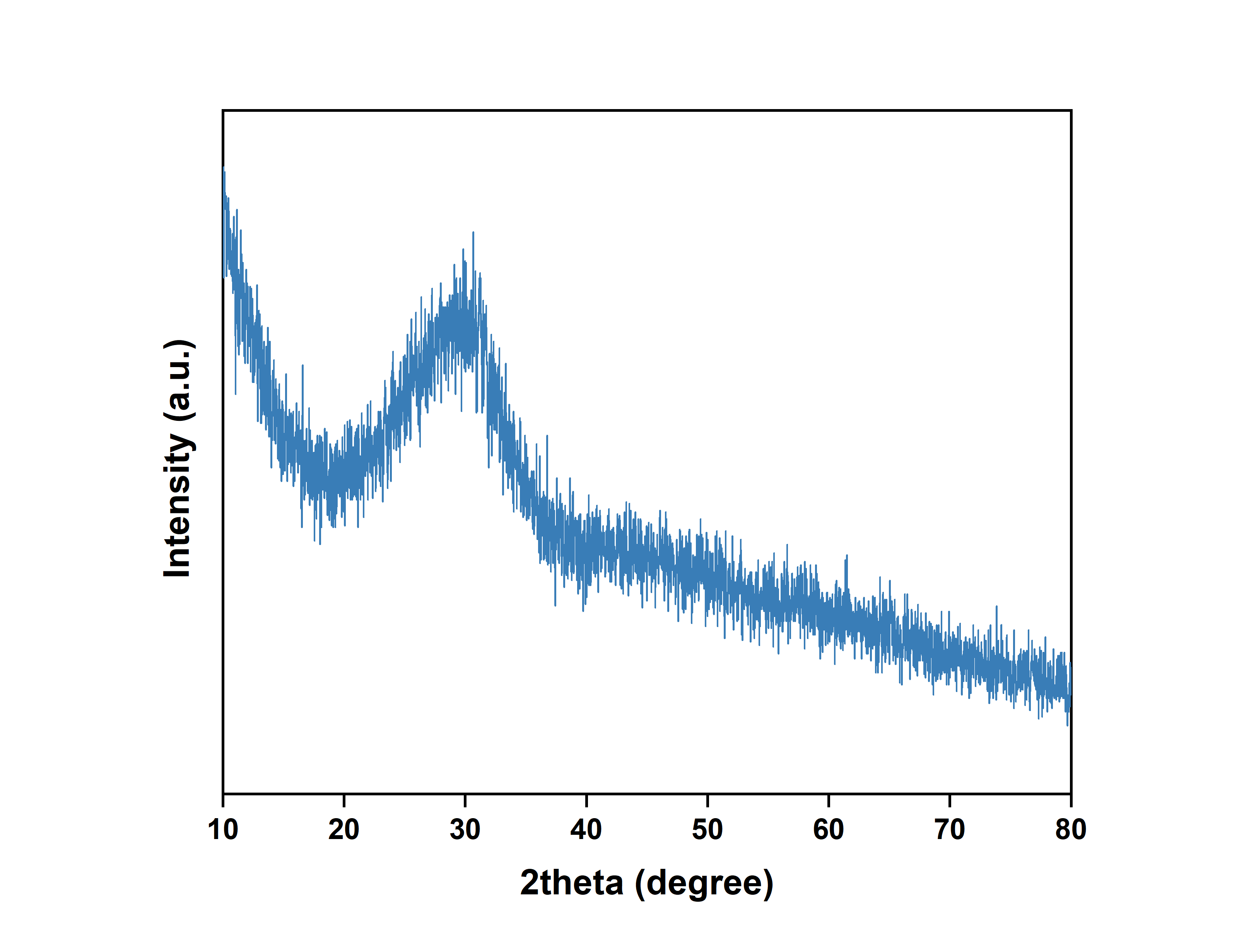


Figure S2. The cross-section image of (6RE_1/6_)TaO_4_ corrode by CMAS at 1200 °C for 10 min.


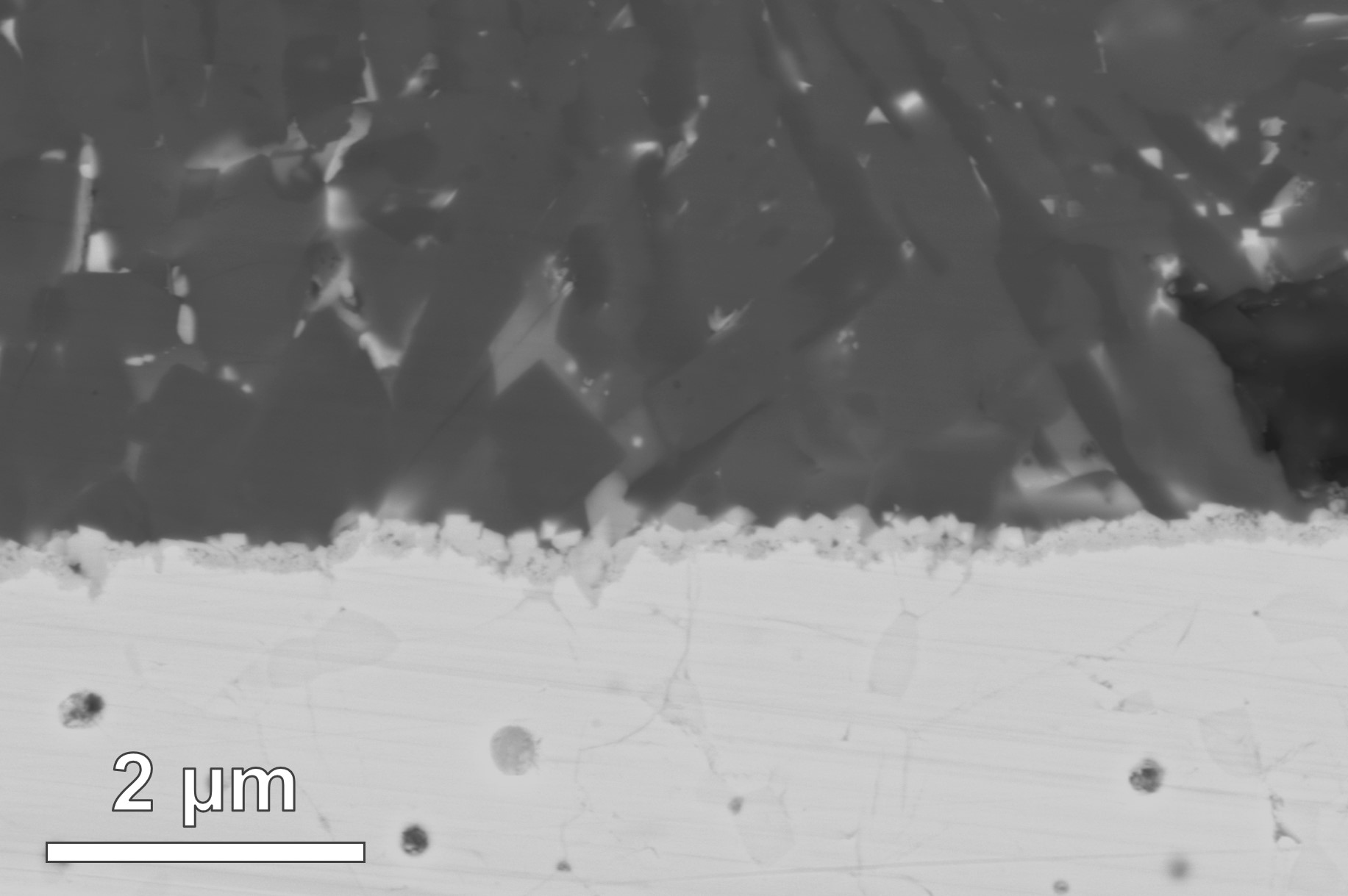


Figure S3. Apatite, a non-dominant CMAS corrosion product of RETaO_4_. a) The cross-section image of (6RE_1/6_)TaO_4_ and (9RE_1/9_)TaO_4_ corrode by CMAS at 1300 °C for 10 h, b) Element compositions of apatite.


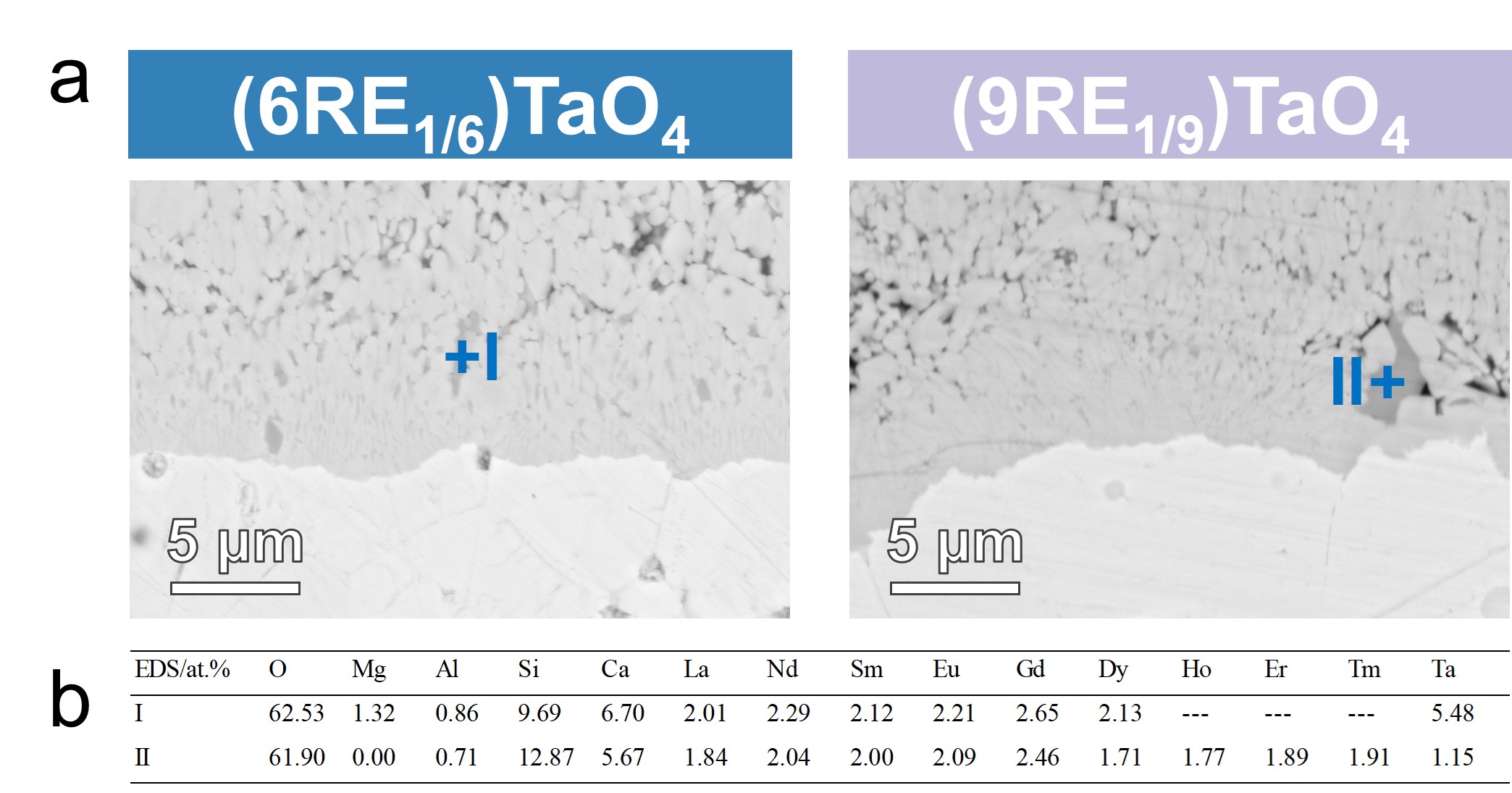


Table S1. Element compositions of various points presented in Figure 1c.

| EDS/at.% | O | La | Nd | Sm | Eu | Gd | Dy | Ho | Er | Tm | Yb | Ta |
| --- | --- | --- | --- | --- | --- | --- | --- | --- | --- | --- | --- | --- |
| I | 66.75 | 5.06 | 5.50 | 5.83 | --- | --- | --- | --- | --- | --- | --- | 16.87 |
| II | 66.66 | 4.10 | 4.41 | 3.91 | 4.28 | --- | --- | --- | --- | --- | --- | 16.65 |
| III | 66.68 | 3.23 | 3.30 | 3.06 | 3.31 | 3.72 | --- | --- | --- | --- | --- | 16.70 |
| IV | 66.70 | 2.48 | 2.64 | 2.55 | 2.85 | 3.13 | 2.90 | --- | --- | --- | --- | 16.75 |
| V | 66.64 | 2.03 | 2.43 | 2.41 | 2.59 | 2.71 | 2.19 | 2.42 | --- | --- | --- | 16.59 |
| VI | 66.72 | 1.74 | 2.03 | 2.22 | 2.26 | 2.31 | 2.05 | 1.94 | 1.94 | --- | --- | 16.80 |
| VII | 66.82 | 1.48 | 1.77 | 1.96 | 1.90 | 2.01 | 1.53 | 1.41 | 1.75 | 2.32 | --- | 17.05 |
| VIII | 66.74 | 1.59 | 1.73 | 1.66 | 1.70 | 1.90 | 1.43 | 1.13 | 1.51 | 1.77 | 1.99 | 16.84 |

Table S2. Element compositions of various points presented in Figure 2b-d.

| EDS/at.% | O | Mg | Al | Si | Ca | La | Nd | Sm | Eu | Gd | Dy | Ta |
| --- | --- | --- | --- | --- | --- | --- | --- | --- | --- | --- | --- | --- |
| I | 66.47 | --- | --- | --- | --- | 2.66 | 2.59 | 2.87 | 2.94 | 3.32 | 2.98 | 16.17 |
| II | 60.51 | 1.92 | 22.02 | 10.01 | 5.54 | --- | --- | --- | --- | --- | --- | --- |
| III | 60.63 | 1.84 | 4.79 | 15.31 | 13.49 | 0.43 | 0.40 | 0.39 | 0.38 | 0.41 | 0.35 | 1.59 |
| IV | 63.21 | 1.88 | 2.58 | 6.34 | 9.38 | 0.44 | 0.96 | 1.10 | 1.25 | 1.20 | 1.14 | 10.50 |
| V | 61.51 | 0.82 | 2.01 | 14.43 | 7.59 | 1.95 | 2.15 | 1.99 | 2.19 | 2.55 | 2.05 | 0.76 |
| VI | 61.05 | 1.41 | 4.78 | 16.34 | 12.61 | 0.56 | 0.38 | 0.32 | 0.33 | 0.40 | 0.35 | 1.46 |
| VII | 63.26 | 1.14 | 3.00 | 9.20 | 9.94 | 0.49 | 0.65 | 0.72 | 0.86 | 0.86 | 0.76 | 9.10 |
| VIII | 61.20 | 2.05 | 2.44 | 15.83 | 8.44 | 1.45 | 1.58 | 1.63 | 1.62 | 1.86 | 1.58 | 0.33 |

Table S3. Element compositions of various points presented in Figure 3m.

| EDS/at.% | O | Mg | Al | Si | Ca | La | Nd | Sm | Eu | Gd | Dy | Ta |
| --- | --- | --- | --- | --- | --- | --- | --- | --- | --- | --- | --- | --- |
| I | 60.44 | 4.45 | 5.53 | 16.63 | 11.32 | 0.18 | 0.16 | 0.16 | 0.16 | 0.12 | 0.16 | 0.68 |
| II | 63.60 | 2.74 | 1.05 | 3.94 | 8.08 | 0.80 | 1.32 | 1.29 | 1.67 | 1.67 | 1.39 | 12.45 |
| III | 66.64 | --- | --- | --- | --- | 2.69 | 2.77 | 2.37 | 2.75 | 3.37 | 2.82 | 16.60 |

Table S4. Element compositions of various points presented in Figure 6.

| EDS/at.% | | O | Mg | Al | Si | Ca | La | Nd | Sm | Eu | Gd | Dy | Ho | Er | Tm | Yb | Ta |
| --- | --- | --- | --- | --- | --- | --- | --- | --- | --- | --- | --- | --- | --- | --- | --- | --- | --- |
| 3 | I | 61.15 | 3.31 | 4.39 | 16.63 | 10.48 | 1.08 | 0.78 | 0.71 | --- | --- | --- | --- | --- | --- | --- | 1.46 |
|  | II | 63.73 | 2.36 | 1.25 | 4.22 | 8.56 | 1.40 | 2.76 | 3.05 | --- | --- | --- | --- | --- | --- | --- | 12.67 |
|  | III | 66.65 | --- | --- | --- | --- | 5.18 | 5.88 | 5.66 | --- | --- | --- | --- | --- | --- | --- | 16.63 |
| 4 | I | 61.15 | 3.41 | 4.35 | 16.89 | 10.39 | 0.86 | 0.58 | 0.48 | 0.52 | --- | --- | --- | --- | --- | --- | 1.34 |
|  | II | 63.90 | 2.27 | 0.62 | 2.72 | 9.35 | 1.62 | 1.93 | 1.69 | 1.71 | --- | --- | --- | --- | --- | --- | 14.19 |
|  | III | 66.65 | --- | --- | --- | --- | 4.18 | 3.99 | 4.01 | 4.54 |  | --- | --- | --- | --- | --- | 16.62 |
| 5 | I | 61.08 | 3.45 | 4.49 | 16.97 | 10.62 | 0.57 | 0.34 | 0.37 | 0.38 | 0.51 | --- | --- | --- | --- | --- | 1.24 |
|  | II | 64.05 | 2.02 | 1.06 | 3.12 | 8.22 | 0.92 | 1.60 | 1.67 | 1.77 | 1.90 | --- | --- | --- | --- | --- | 13.67 |
|  | III | 66.62 | --- | --- | --- | --- | 3.09 | 3.41 | 3.36 | 2.89 | 4.07 | --- | --- | --- | --- | --- | 16.56 |
| 6 | I | 61.03 | 3.65 | 4.42 | 17.05 | 10.58 | 0.44 | 0.35 | 0.31 | 0.35 | 0.38 | 0.27 | --- | --- | --- | --- | 1.18 |
|  | II | 63.80 | 2.08 | 1.36 | 4.15 | 8.17 | 0.59 | 1.35 | 1.36 | 1.78 | 1.59 | 1.24 | --- | --- | --- | --- | 12.54 |
|  | III | 66.58 | --- | --- | --- | --- | 2.61 | 2.64 | 2.78 | 2.95 | 3.04 | 2.95 | --- | --- | --- | --- | 16.44 |
| 7 | I | 57.54 | 4.02 | 4.94 | 18.34 | 11.89 | 0.44 | 0.29 | 0.31 | 0.29 | 0.35 | 0.22 | 0.28 | --- | --- | --- | 1.10 |
|  | II | 63.69 | 2.06 | 1.44 | 3.19 | 8.49 | 0.54 | 1.19 | 1.32 | 1.48 | 1.49 | 1.17 | 1.05 | --- | --- | --- | 12.89 |
|  | III | 67.00 | --- | --- | --- | --- | 2.13 | 2.23 | 2.29 | 2.30 | 2.69 | 1.97 | 1.89 | --- | --- | --- | 17.50 |
| 8 | I | 60.77 | 3.67 | 4.60 | 16.60 | 11.38 | 0.27 | 0.25 | 0.19 | 0.22 | 0.23 | 0.23 | 0.21 | 0.24 | --- | --- | 1.15 |
|  | II | 64.02 | 1.72 | 1.12 | 3.31 | 7.95 | 0.65 | 1.07 | 1.16 | 1.31 | 1.44 | 1.12 | 0.93 | 0.96 | --- | --- | 13.24 |
|  | III | 66.70 | --- | --- | --- | --- | 1.76 | 2.03 | 2.18 | 2.24 | 2.49 | 1.89 | 2.00 | 1.95 | --- | --- | 16.76 |
| 9 | I | 60.67 | 3.86 | 4.17 | 16.55 | 11.62 | 0.30 | 0.21 | 0.23 | 0.24 | 0.29 | 0.20 | 0.14 | 0.20 | 0.19 | --- | 1.13 |
|  | II | 63.81 | 1.96 | 1.40 | 3.50 | 7.84 | 0.47 | 0.93 | 1.08 | 1.16 | 1.32 | 0.89 | 0.86 | 0.96 | 1.14 | --- | 12.68 |
|  | III | 66.72 | --- | --- | --- | --- | 1.55 | 1.83 | 1.83 | 2.06 | 2.21 | 1.43 | 1.55 | 1.76 | 2.24 | --- | 16.81 |
| 10 | I | 60.77 | 3.74 | 4.30 | 16.73 | 11.33 | 0.28 | 0.23 | 0.22 | 0.21 | 0.17 | 0.15 | 0.16 | 0.23 | 0.23 | 0.19 | 1.09 |
|  | II | 63.80 | 1.66 | 1.71 | 3.66 | 7.58 | 0.57 | 0.96 | 0.93 | 1.11 | 1.18 | 0.89 | 0.72 | 0.79 | 1.03 | 1.14 | 12.27 |
|  | III | 66.67 | --- | --- | --- | --- | 1.42 | 1.50 | 1.52 | 1.72 | 1.96 | 1.63 | 1.36 | 1.58 | 1.94 | 2.02 | 16.68 |

Table S5. Element compositions of various points presented in Figure 8c.

| EDS/at.% | O | Mg | Al | Si | Ca | La | Nd | Sm | Eu | Ta |
| --- | --- | --- | --- | --- | --- | --- | --- | --- | --- | --- |
| I | 63.29 | 2.50 | 3.18 | 15.43 | 4.36 | 2.94 | 1.63 | 1.33 | 1.41 | 3.94 |
| II | 64.09 | 2.35 | 1.01 | 2.57 | 7.63 | 1.71 | 2.21 | 2.03 | 2.49 | 13.92 |
| III | 64.40 | 2.16 | 1.03 | 2.72 | 6.68 | 1.81 | 2.38 | 2.25 | 2.51 | 14.06 |
| IV | 61.92 | 0.08 | 0.01 | 13.72 | 4.81 | 6.52 | 4.79 | 3.87 | 3.90 | 0.38 |
| V | 66.78 | --- | --- | --- | --- | 3.92 | 3.98 | 4.00 | 4.38 | 16.95 |

Table S6. Element compositions of various points presented in Figure 9b.

| EDS/at.% | O | Mg | Al | Si | Ca | La | Nd | Sm | Eu | Gd | Ta |
| --- | --- | --- | --- | --- | --- | --- | --- | --- | --- | --- | --- |
| I | 58.48 | 3.54 | 4.81 | 19.80 | 9.70 | 1.03 | 0.57 | 0.47 | 0.42 | 0.54 | 0.64 |
| II | 63.96 | 2.11 | 1.08 | 2.91 | 7.73 | 1.11 | 1.77 | 1.88 | 2.08 | 2.02 | 13.36 |
| III | 64.03 | 2.09 | 1.32 | 2.59 | 7.52 | 1.01 | 1.81 | 1.85 | 2.08 | 2.12 | 13.58 |
| IV | 63.23 | 0.22 | 0.00 | 11.57 | 4.33 | 4.91 | 3.30 | 2.63 | 2.43 | 2.83 | 4.55 |
| V | 66.69 | --- | --- | --- | --- | 3.36 | 3.58 | 3.02 | 3.12 | 3.50 | 16.73 |

Table S7. Statistical results on the element compositions of pyrochlore formed from eight RETaO_4_.

| EDS/at.% | | O | Mg | Al | Si | Ca | La | Nd | Sm | Eu | Gd | Dy | Ho | Er | Tm | Yb | Ta |
| --- | --- | --- | --- | --- | --- | --- | --- | --- | --- | --- | --- | --- | --- | --- | --- | --- | --- |
| 3 | I | 63.73 | 2.36 | 1.25 | 4.22 | 8.56 | 1.40 | 2.76 | 3.05 | --- | --- | --- | --- | --- | --- | --- | 12.67 |
|  | II | 63.94 | 2.28 | 0.78 | 2.47 | 8.78 | 1.67 | 2.89 | 3.02 | --- | --- | --- | --- | --- | --- | --- | 14.15 |
|  | III | 63.82 | 2.55 | 0.53 | 2.90 | 8.94 | 2.01 | 2.71 | 2.69 | --- | --- | --- | --- | --- | --- | --- | 13.86 |
| 4 | I | 63.90 | 2.27 | 0.62 | 2.72 | 9.35 | 1.62 | 1.93 | 1.69 | 1.71 | --- | --- | --- | --- | --- | --- | 14.19 |
|  | II | 63.77 | 2.24 | 0.99 | 2.80 | 9.26 | 1.33 | 1.98 | 1.83 | 2.04 | --- | --- | --- | --- | --- | --- | 13.77 |
|  | III | 63.83 | 2.44 | 1.08 | 2.84 | 8.27 | 1.21 | 2.10 | 1.93 | 2.80 | --- | --- | --- | --- | --- | --- | 13.50 |
| 5 | I | 64.05 | 2.02 | 1.06 | 3.12 | 8.22 | 0.92 | 1.60 | 1.67 | 1.77 | 1.90 | --- | --- | --- | --- | --- | 13.67 |
|  | II | 63.89 | 2.05 | 1.19 | 3.39 | 8.32 | 0.85 | 1.66 | 1.79 | 1.81 | 1.86 | --- | --- | --- | --- | --- | 13.21 |
|  | III | 63.91 | 2.14 | 1.00 | 3.68 | 8.19 | 0.78 | 1.55 | 1.67 | 1.90 | 2.07 | --- | --- | --- | --- | --- | 13.11 |
| 6 | I | 63.78 | 2.23 | 1.13 | 3.70 | 8.26 | 0.66 | 1.24 | 1.41 | 1.71 | 1.60 | 1.44 | --- | --- | --- | --- | 12.85 |
|  | II | 63.75 | 2.37 | 1.16 | 3.61 | 8.20 | 0.65 | 1.34 | 1.33 | 1.60 | 1.73 | 1.38 | --- | --- | --- | --- | 12.86 |
|  | III | 63.80 | 2.08 | 1.36 | 4.15 | 8.17 | 0.59 | 1.35 | 1.36 | 1.78 | 1.59 | 1.24 | --- | --- | --- | --- | 12.54 |
| 7 | I | 63.69 | 2.06 | 1.44 | 3.19 | 8.49 | 0.54 | 1.19 | 1.32 | 1.48 | 1.49 | 1.17 | 1.05 | --- | --- | --- | 12.89 |
|  | II | 63.75 | 2.30 | 1.43 | 3.59 | 7.90 | 0.62 | 1.25 | 1.28 | 1.43 | 1.45 | 1.18 | 1.14 | --- | --- | --- | 12.67 |
|  | III | 63.70 | 2.04 | 1.48 | 3.46 | 8.20 | 0.60 | 1.17 | 1.34 | 1.43 | 1.53 | 1.09 | 1.34 | --- | --- | --- | 12.63 |
| 8 | I | 64.02 | 1.72 | 1.12 | 3.31 | 7.95 | 0.65 | 1.07 | 1.16 | 1.31 | 1.44 | 1.12 | 0.93 | 0.96 | --- | --- | 13.24 |
|  | II | 63.98 | 2.06 | 1.20 | 3.25 | 8.04 | 0.48 | 1.04 | 1.14 | 1.26 | 1.42 | 0.97 | 0.83 | 0.97 | --- | --- | 13.37 |
|  | III | 63.87 | 1.86 | 1.49 | 3.15 | 8.05 | 0.52 | 1.10 | 1.16 | 1.19 | 1.31 | 1.17 | 0.99 | 1.07 | --- | --- | 13.06 |
| 9 | I | 63.87 | 1.85 | 2.00 | 2.69 | 7.11 | 0.64 | 1.02 | 1.16 | 1.27 | 1.40 | 1.02 | 0.90 | 0.96 | 1.33 | --- | 12.80 |
|  | II | 63.81 | 1.96 | 1.40 | 3.50 | 7.84 | 0.47 | 0.93 | 1.08 | 1.16 | 1.32 | 0.89 | 0.86 | 0.96 | 1.14 | --- | 12.68 |
|  | III | 63.86 | 1.84 | 0.87 | 4.25 | 7.26 | 0.63 | 1.09 | 1.13 | 1.27 | 1.40 | 1.01 | 0.86 | 1.00 | 1.43 | --- | 12.08 |
| 10 | I | 63.80 | 1.66 | 1.71 | 3.66 | 7.58 | 0.57 | 0.96 | 0.93 | 1.11 | 1.18 | 0.89 | 0.72 | 0.79 | 1.03 | 1.14 | 12.27 |
|  | II | 63.96 | 1.58 | 1.80 | 3.53 | 7.52 | 0.49 | 0.84 | 0.90 | 0.96 | 0.96 | 0.92 | 0.81 | 0.75 | 1.16 | 1.14 | 12.68 |
|  | III | 63.98 | 2.32 | 0.30 | 3.45 | 7.40 | 0.60 | 0.98 | 0.90 | 1.03 | 1.16 | 0.91 | 0.86 | 0.87 | 0.98 | 1.17 | 13.09 |

Table S8. Ionic radii of different elements in the case of 6-coordination and 8-coordination.

| Element | [AO8]/Å | [BO6]/Å |
| --- | --- | --- |
| Mg | 0.890 | 0.720 |
| Al | --- | 0.535 |
| Si | --- | 0.400 |
| Ca | 1.120 | 1.000 |
| La | 1.160 | 1.032 |
| Nd | 1.109 | 0.983 |
| Sm | 1.079 | 0.958 |
| Eu | 1.066 | 0.947 |
| Gd | 1.053 | 0.938 |
| Dy | 1.027 | 0.912 |
| Ho | 1.015 | 0.901 |
| Er | 1.004 | 0.890 |
| Tm | 0.994 | 0.880 |
| Yb | 0.985 | 0.868 |
| Ta | 0.740 | 0.640 |

Table S9. Statistical results on the element compositions of residual CMAS from eight RETaO_4_.

| EDS/at.% | | O | Mg | Al | Si | Ca | La | Nd | Sm | Eu | Gd | Dy | Ho | Er | Tm | Yb | Ta |
| --- | --- | --- | --- | --- | --- | --- | --- | --- | --- | --- | --- | --- | --- | --- | --- | --- | --- |
| 3 | I | 63.73 | 2.36 | 1.25 | 4.22 | 8.56 | 1.40 | 2.76 | 3.05 | --- | --- | --- | --- | --- | --- | --- | 12.67 |
|  | II | 63.94 | 2.28 | 0.78 | 2.47 | 8.78 | 1.67 | 2.89 | 3.02 | --- | --- | --- | --- | --- | --- | --- | 14.15 |
|  | III | 63.82 | 2.55 | 0.53 | 2.90 | 8.94 | 2.01 | 2.71 | 2.69 | --- | --- | --- | --- | --- | --- | --- | 13.86 |
| 4 | I | 63.90 | 2.27 | 0.62 | 2.72 | 9.35 | 1.62 | 1.93 | 1.69 | 1.71 | --- | --- | --- | --- | --- | --- | 14.19 |
|  | II | 63.77 | 2.24 | 0.99 | 2.80 | 9.26 | 1.33 | 1.98 | 1.83 | 2.04 | --- | --- | --- | --- | --- | --- | 13.77 |
|  | III | 63.83 | 2.44 | 1.08 | 2.84 | 8.27 | 1.21 | 2.10 | 1.93 | 2.80 | --- | --- | --- | --- | --- | --- | 13.50 |
| 5 | I | 64.05 | 2.02 | 1.06 | 3.12 | 8.22 | 0.92 | 1.60 | 1.67 | 1.77 | 1.90 | --- | --- | --- | --- | --- | 13.67 |
|  | II | 63.89 | 2.05 | 1.19 | 3.39 | 8.32 | 0.85 | 1.66 | 1.79 | 1.81 | 1.86 | --- | --- | --- | --- | --- | 13.21 |
|  | III | 63.91 | 2.14 | 1.00 | 3.68 | 8.19 | 0.78 | 1.55 | 1.67 | 1.90 | 2.07 | --- | --- | --- | --- | --- | 13.11 |
| 6 | I | 63.78 | 2.23 | 1.13 | 3.70 | 8.26 | 0.66 | 1.24 | 1.41 | 1.71 | 1.60 | 1.44 | --- | --- | --- | --- | 12.85 |
|  | II | 63.75 | 2.37 | 1.16 | 3.61 | 8.20 | 0.65 | 1.34 | 1.33 | 1.60 | 1.73 | 1.38 | --- | --- | --- | --- | 12.86 |
|  | III | 63.80 | 2.08 | 1.36 | 4.15 | 8.17 | 0.59 | 1.35 | 1.36 | 1.78 | 1.59 | 1.24 | --- | --- | --- | --- | 12.54 |
| 7 | I | 63.69 | 2.06 | 1.44 | 3.19 | 8.49 | 0.54 | 1.19 | 1.32 | 1.48 | 1.49 | 1.17 | 1.05 | --- | --- | --- | 12.89 |
|  | II | 63.75 | 2.30 | 1.43 | 3.59 | 7.90 | 0.62 | 1.25 | 1.28 | 1.43 | 1.45 | 1.18 | 1.14 | --- | --- | --- | 12.67 |
|  | III | 63.70 | 2.04 | 1.48 | 3.46 | 8.20 | 0.60 | 1.17 | 1.34 | 1.43 | 1.53 | 1.09 | 1.34 | --- | --- | --- | 12.63 |
| 8 | I | 64.02 | 1.72 | 1.12 | 3.31 | 7.95 | 0.65 | 1.07 | 1.16 | 1.31 | 1.44 | 1.12 | 0.93 | 0.96 | --- | --- | 13.24 |
|  | II | 63.98 | 2.06 | 1.20 | 3.25 | 8.04 | 0.48 | 1.04 | 1.14 | 1.26 | 1.42 | 0.97 | 0.83 | 0.97 | --- | --- | 13.37 |
|  | III | 63.87 | 1.86 | 1.49 | 3.15 | 8.05 | 0.52 | 1.10 | 1.16 | 1.19 | 1.31 | 1.17 | 0.99 | 1.07 | --- | --- | 13.06 |
| 9 | I | 63.87 | 1.85 | 2.00 | 2.69 | 7.11 | 0.64 | 1.02 | 1.16 | 1.27 | 1.40 | 1.02 | 0.90 | 0.96 | 1.33 | --- | 12.80 |
|  | II | 63.81 | 1.96 | 1.40 | 3.50 | 7.84 | 0.47 | 0.93 | 1.08 | 1.16 | 1.32 | 0.89 | 0.86 | 0.96 | 1.14 | --- | 12.68 |
|  | III | 63.86 | 1.84 | 0.87 | 4.25 | 7.26 | 0.63 | 1.09 | 1.13 | 1.27 | 1.40 | 1.01 | 0.86 | 1.00 | 1.43 | --- | 12.08 |
| 10 | I | 63.80 | 1.66 | 1.71 | 3.66 | 7.58 | 0.57 | 0.96 | 0.93 | 1.11 | 1.18 | 0.89 | 0.72 | 0.79 | 1.03 | 1.14 | 12.27 |
|  | II | 63.96 | 1.58 | 1.80 | 3.53 | 7.52 | 0.49 | 0.84 | 0.90 | 0.96 | 0.96 | 0.92 | 0.81 | 0.75 | 1.16 | 1.14 | 12.68 |
|  | III | 63.98 | 2.32 | 0.30 | 3.45 | 7.40 | 0.60 | 0.98 | 0.90 | 1.03 | 1.16 | 0.91 | 0.86 | 0.87 | 0.98 | 1.17 | 13.09 |
